# Comparison of dynamic updating strategies for clinical prediction models

**DOI:** 10.1186/s41512-021-00110-w

**Published:** 2021-12-06

**Authors:** Erin M. Schnellinger, Wei Yang, Stephen E. Kimmel

**Affiliations:** 1 Department of Biostatistics, Epidemiology, and Informatics, Perelman School of Medicine, University of Pennsylvania, Philadelphia, PA USA; 2 Department of Epidemiology, College of Public Health and Health Professions and College of Medicine, University of Florida, 2004 Mowry Road, Gainesville, FL 32610 USA

**Keywords:** Prediction model updating, Recalibration, Model revision, Closed testing procedure

## Abstract

**Background:**

Prediction models inform many medical decisions, but their performance often deteriorates over time. Several discrete-time update strategies have been proposed in the literature, including model recalibration and revision. However, these strategies have not been compared in the dynamic updating setting.

**Methods:**

We used post-lung transplant survival data during 2010-2015 and compared the Brier Score (BS), discrimination, and calibration of the following update strategies: (1) never update, (2) update using the closed testing procedure proposed in the literature, (3) always recalibrate the intercept, (4) always recalibrate the intercept and slope, and (5) always refit/revise the model. In each case, we explored update intervals of every 1, 2, 4, and 8 quarters. We also examined how the performance of the update strategies changed as the amount of old data included in the update (i.e., sliding window length) increased.

**Results:**

All methods of updating the model led to meaningful improvement in BS relative to never updating. More frequent updating yielded better BS, discrimination, and calibration, regardless of update strategy. Recalibration strategies led to more consistent improvements and less variability over time compared to the other updating strategies. Using longer sliding windows did not substantially impact the recalibration strategies, but did improve the discrimination and calibration of the closed testing procedure and model revision strategies.

**Conclusions:**

Model updating leads to improved BS, with more frequent updating performing better than less frequent updating. Model recalibration strategies appeared to be the least sensitive to the update interval and sliding window length.

**Supplementary Information:**

The online version contains supplementary material available at 10.1186/s41512-021-00110-w.

## Background

Prediction models inform many medical decisions, but their performance often deteriorates over time due to changes in underlying clinical populations or evolving medical practices. Unfortunately, many prediction models are static; that is, they are developed once (i.e., at a single time point) within a fixed derivation cohort and then tested on new (future) members of this cohort, who may differ in clinical characteristics or disease risk. In contrast, dynamic prediction modeling involves updating models at multiple time points as new data are accrued [1, 2]. Several discrete-time update strategies have been proposed in the literature, including recalibration of the intercept, recalibration of the intercept and slope, and model revision [1–8].

Some of these strategies have been evaluated as methods for updating a model at a single time point [3–5, 9]. However, they have not been rigorously tested or compared when applied to dynamic changes in models over time [2]. For example, while Hickey et al. [10] examined the change in model coefficients when models were refitted in dynamic settings, how actual model predictive performance changes over time remains unknown, both for refitting strategies and when comparing alternative updating strategies (e.g., recalibration). Similarly, Van Calster et al. developed methods to validate and update multinomial logistic regression prediction models, and presented an empiric example in which they compared the predictive performance of a model which was updated at a single timepoint using more recent data from the same setting (i.e., temporal updating) and using data from a different setting (i.e., geographic updating) [9]. How their methods might perform in dynamic updating settings is still unclear [9]. Minne et al. examined repeated recalibration of decision-trees and logistic regression models, but did not compare these methods to other model updating strategies [11]. Finally, the data-driven testing approach to model updating over time proposed by Davis et al. [12] indicates that different update methods require different frequencies of updating (i.e., update interval) [12], but more research is needed to understand how the size of this update interval and the amount of old versus new data used for each update (i.e., the sliding window) might influence model performance.

In this paper, we compare several previously proposed update strategiesbroadly classified as never update, update based on a series of statistical tests comparing candidate update models to the current model at each update interval (see Additional file), or update following a pre-specified schedule—in a time-dependent manner, using the empiric example of predicting 1-year post-lung transplant survival. Consistent with Hickey et al. [10] and Jenkins et al. [2], we refer to these strategies as “dynamic updating strategies” to reflect the fact that they are applied in a time-dependent manner. In the USA, 1-year post-lung transplant survival is used both to assist in allocating donor organs to lung transplant candidates (through the Lung Allocation Score, LAS) and to evaluate the performance of transplant programs [13]. However, the model is not updated frequently. Since its adoption in May 2005, the LAS has only been updated two times—in 2010 and 2015 [14]—despite a growing body of research suggesting that the clinical characteristics of transplant patients have evolved over time toward older and sicker patients [15] and the changing surgical methods and care of patients undergoing transplantation. Here, we examine what would have happened had the 1-year post-lung transplant survival model been updated more frequently between 2010 and 2015 under various update strategies, update intervals, and length of sliding windows.

## Methods

This study used post-lung transplant data from the United Network for Organ Sharing (UNOS) transplant registry during 2007-2015. Patients were included in our study if they were 18 years or older and received a single or bi-lateral lung transplant in the USA during this time. Patients were excluded if they were registered for multiple-organ transplant (e.g., heart-lung). These data were partitioned into baseline and post-baseline periods, with the baseline period including data between 2007 and 2009, and the post-baseline period including data between 2010 and 2015. The baseline prediction model was set by fitting a logistic regression model for 1-year post-lung transplant mortality using the baseline period data and the same covariates as the 2010 post-transplant LAS model (i.e., age, cardiac index, continuous mechanical ventilation, serum creatinine, diagnosis group, functional status, oxygen need at rest, and 6-min walk distance) [13].

Subsequently, five different update strategies were examined: (1) never update, (2) update using the closed testing procedure proposed by Vergouwe et al. [5], (3) always recalibrate the intercept [1–8], (4) always recalibrate the intercept and slope [1–8], and (5) always refit the model (model revision [5, 6]). Methodologic details of each of these strategies appear in the Additional file.

**Fig. 1:**
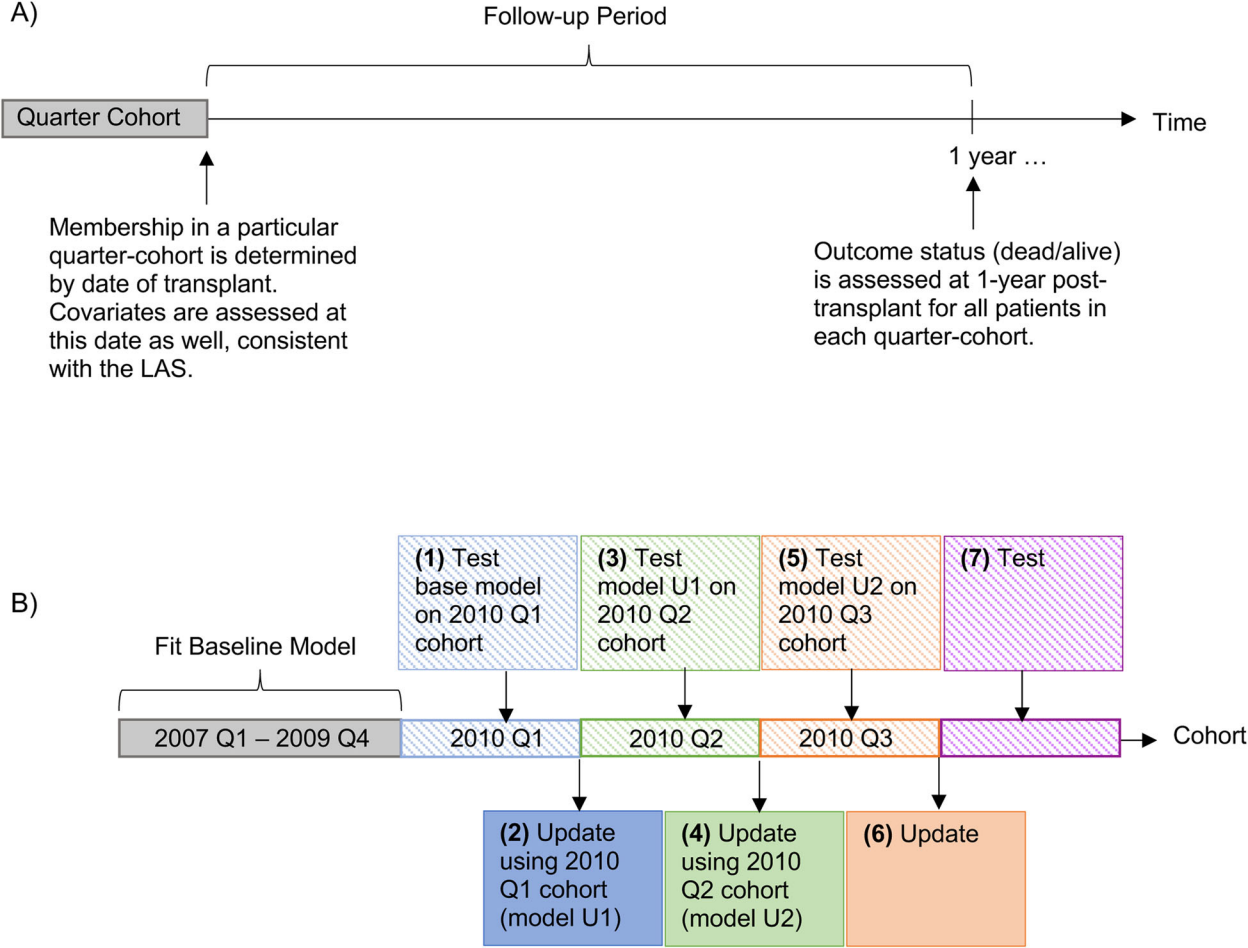
Illustration of how quarter-cohorts were defined and used to construct baseline and updated prediction models. **A** Individuals were assigned to a particular quarter-cohort based on their date of transplant and followed for 1 year, similar to an “incident-user cohort” design [16]. **B** (1) The baseline model was developed using data from individuals who were transplanted between 2007 and 2009 (i.e., the quarter-cohorts between 2007 and 2009) and tested on the 2010 quarter 1 (Q1) cohort; (2) the baseline model then was updated using the 2010 Q1 cohort data according to each of the five different update strategies (model U1); (3) model U1 then was tested on the 2010 Q2 cohort; (4) model U1 was updated using the 2010 Q2 cohort data according to each of the five different update strategies (model U2); (5) model U2 was tested on the 2010 Q3 cohort; (6) and (7) subsequent updates occur in the same fashion. A similar procedure was used when the update interval was increased to 2, 4, and 8 quarter-cohorts

These five strategies were initially implemented every quarter, using just the most recent quarter of data. Quarters were defined based on the date each patient received transplant, and all follow-up data for each patient were included in the quarter associated with that patient’s transplant date (i.e., each quarter is actually an “incident-cohort” [16] of patients who received transplant in that quarter; see Fig. 1A). Outcomes were assessed at 1-year post-transplant for all patients in each quarter-cohort.

Model updating proceeded as shown in Fig. 1B. First, the baseline model (which was developed using quarter-cohorts between 2007 and 2009) was tested on the 2010 Q1 cohort; second, the baseline model was updated using the 2010 Q1 cohort data according to each of the five different update strategies (model U1); third, model U1 then was tested on the 2010 Q2 cohort; fourth, model U1 was updated using the 2010 Q2 cohort data according to each of the five different update strategies (model U2); and fifth, model U2 was tested on the 2010 Q3 cohort. Subsequent updates occurred on top of previous update(s), and proceeded in the same fashion (i.e., models were updated using data from the current quarter-cohort and tested on data from the next quarter-cohort).

The primary measure was the overall performance of each update strategy, evaluated by computing the Brier score (BS) both for each quarter separately and across all quarters in the post-baseline data. Since we did not use the same data to develop and test our models (i.e., models were fit in one quarter-cohort and then tested on the next cohort of new patients), over-optimism was not a concern. Improvement in BS was calculated at each quarter as the difference in BS between each update strategy and never update. Discrimination was assessed across all quarters via C-statistics (i.e., the area under the receiver operating characteristic curve, AUC) and calibration was assessed via Hosmer-Lemeshow (H-L) statistics, calibration intercepts, and calibration slopes. We also computed the difference in BS between pairs of strategies at each individual quarter in the post-baseline data and performed Wilcoxon signed rank tests to determine which update strategy performed best.

We examined how the performance of these update strategies changed when the size of the update interval and length of the sliding window increased. Specifically, we investigated update intervals of every 1, 2, 4, and 8 quarters. Under each update interval, updates were performed by using all data accrued since the most recent update (i.e., all new data). Thus, when the update interval equaled 1, 2, 4, and 8 quarters, we used 1 quarter new, 2 quarters new, 4 quarters new, and 8 quarters new data to perform each update, respectively. In the sliding window analysis, the update interval was fixed at 1 quarter, and the following sliding windows were considered: 1 quarter new (100% new), 1 quarter new + 1 quarter old (50% new, 50% old), 1 quarter new + 3 quarters old (25% new, 75% old), and 1 quarter new + 7 quarters old (12.5% new, 87.5% old). All analyses were conducted using R (R Foundation for Statistical Computing, Vienna, Austria).

## Results

### Sample size and mortality risk

The baseline-period contained 2853 patients and 508 events (deaths), translating to an overall mortality risk of 0.178. The post-baseline period contained 10,948 patients and 1449 events, translating to an overall mortality risk of 0.132. When examined per quarter, the mean (standard deviation) sample size in the post-baseline period was 456.2 (32.6) patients per quarter, and on average, 60.4 (8.8) events occurred per quarter (Additional file 1: Table 1). Thus, the observed mean mortality risk per quarter in the post-baseline period was 0.133 (0.021). The mortality risk tended to decrease over time (Additional file 1: Fig. 1).

### Examination of updating strategies in reference case

**Fig. 2:**
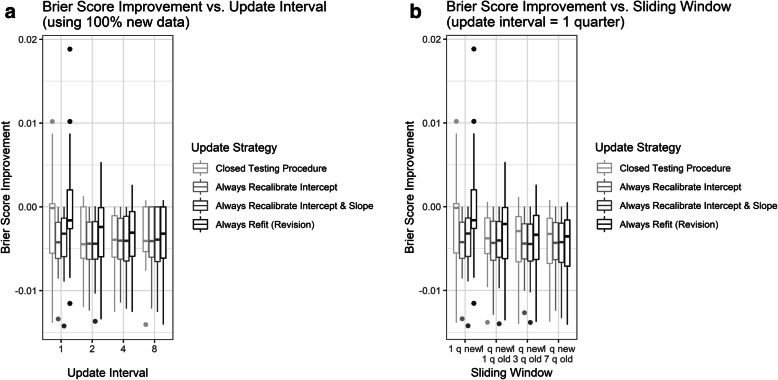
Boxplot of difference in Brier score between each update strategy and never update calculated at each quarter versus (**A**) update interval, when 100% new data are used to update the model; and (**B**) sliding window, when the update interval equals 1 quarter. Horizontal bars represent medians and interquartile ranges (IQR). Negative values reflect improvement compared never update

At an update interval of one quarter with 100% new data (reference case), all methods of updating improved the BS of the model relative to never updating (Fig. 2). The extent of improvement in BS was largest for the recalibration strategies (Fig. 2). The always recalibrate intercept and always recalibrate intercept and slope strategies also exhibited larger (better) AUC (0.605 and 0.601, respectively), smaller (better) H-L (29.33 and 18.53, respectively), calibration intercepts closer to zero (−0.472 and −0.451, respectively) and calibration slopes closer to one (0.758 and 0.771, respectively) than the closed testing procedure and always refit strategies (AUC 0.575 and 0.570, H-L 118.4 and 357.8, calibration intercept −1.658 and −1.835, and calibration slope 0.118 and 0.021, respectively; Tables 1 and 2, Additional file 1: Fig. 2). The always refit/revision strategy exhibited the worst performance in the reference case, regardless of which metric was used. The BS, AUC, H-L, calibration intercept, and calibration slope of the closed testing procedure fell in between those of the recalibration strategies and always refit/revision in the reference case, suggesting intermediate performance. That said, the closed testing procedure was able to attain this performance with half the number of updates (12 updates) compared with the always recalibrate and always refit/revision strategies (24 updates for each).

**Table 1:** Brier score (BS), AUC, Hosmer-Lemeshow statistic, calibration intercept and slope (from logistic calibration), and number of updates performed under each update strategy when the update interval equals 1, 2, 4, and 8 quarters and 100% new data are used

Update interval	Metric	1 quarter	2 quarters	4 quarters	8 quarters
**Never update**	BS	0.118	0.118	0.118	0.118
	AUC	0.603	0.603	0.603	0.603
	H-L	290.5	290.5	290.5	290.5
	Calibration intercept	−0.754	−0.754	−0.754	−0.754
	Calibration slope	0.791	0.791	0.791	0.791
	# Updates	0	0	0	0
**Closed testing procedure**	BS	0.116	0.114	0.114	0.114
	AUC	0.575	0.602	0.598	0.602
	H-L	118.4	54.64	37.80	82.15
	Calibration intercept	−1.658	−0.561	−0.570	−0.887
	Calibration slope	0.118	0.754	0.724	0.577
	# Updates	12	5	4	2
**Always recalibrate intercept**	BS	0.113	0.113	0.114	0.114
	AUC	0.605	0.605	0.603	0.605
	H-L	29.33	28.92	39.21	69.76
	Calibration intercept	−0.472	−0.453	−0.525	−0.595
	Calibration slope	0.758	0.781	0.760	0.755
	# Updates	24	12	6	3
**Always recalibrate intercept and slope**	BS	0.113	0.113	0.114	0.114
	AUC	0.601	0.602	0.602	0.605
	H-L	18.53	27.91	35.41	59.22
	Calibration intercept	−0.451	−0.353	−0.443	−0.517
	Calibration slope	0.771	0.839	0.809	0.802
	# Updates	24	12	6	3
**Always refit (revision)**	BS	0.118	0.114	0.114	0.114
	AUC	0.570	0.582	0.592	0.601
	H-L	357.8	106.6	56.25	77.01
	Calibration intercept	−1.835	−1.534	−0.972	−0.897
	Calibration slope	0.021	0.180	0.499	0.570
	# Updates	24	12	6	3

**Table 2:** Brier score (BS), AUC, Hosmer-Lemeshow statistic, calibration intercept and slope (from logistic calibration), and number of updates performed under each update strategy when the sliding window equals 1, 2, 4, or 8 quarters and the update interval equals 1 quarter

Sliding window	Metric	1 quarter new	2 quarters (1 q new + 1 q old)	4 quarters (1 q new + 3 q old)	8 quarters (1 q new + 7 q old)
**Never update**	BS	0.118	0.118	0.118	0.118
	AUC	0.603	0.603	0.603	0.603
	H-L	290.5	290.5	290.5	290.5
	Calibration intercept	−0.754	−0.754	−0.754	−0.754
	Calibration slope	0.791	0.791	0.791	0.791
	# Updates	0	0	0	0
**Closed testing procedure**	BS	0.116	0.114	0.114	0.114
	AUC	0.575	0.601	0.593	0.600
	H-L	118.4	36.95	34.75	42.47
	Calibration intercept	−1.658	−0.503	−0.829	−0.755
	Calibration slope	0.118	0.766	0.572	0.635
	# Updates	12	8	8	6
**Always recalibrate intercept**	BS	0.113	0.113	0.113	0.113
	AUC	0.605	0.605	0.607	0.608
	H-L	29.33	33.04	25.56	31.61
	Calibration intercept	−0.472	−0.454	−0.414	−0.437
	Calibration slope	0.758	0.773	0.802	0.803
	# Updates	24	24	24	24
**Always recalibrate intercept and slope**	BS	0.113	0.113	0.113	0.113
	AUC	0.601	0.602	0.606	0.607
	H-L	18.53	20.89	18.73	22.15
	Calibration intercept	−0.451	−0.317	−0.218	−0.240
	Calibration slope	0.771	0.852	0.914	0.917
	# Updates	24	24	24	24
**Always refit (revision)**	BS	0.118	0.115	0.114	0.113
	AUC	0.570	0.588	0.594	0.602
	H-L	357.8	81.77	43.42	25.26
	Calibration intercept	−1.835	−1.191	−0.889	−0.504
	Calibration slope	0.021	0.362	0.532	0.761
	# Updates	24	24	24	24

### Varying the update interval

Increasing the length of the update interval did not meaningfully improve the performance of the recalibration strategies and, in fact, tended to lead to worse BS, H-L, and calibration intercepts and slopes (Fig. 2A and Table 1). In contrast, the closed testing procedure and always refit/revision strategies were more sensitive to the update interval length. Specifically, BS and AUC tended to improve under the closed testing procedure and always refit/revision strategies as the update interval increased (i.e., more new data accumulated). Among all strategies, the H-L, calibration intercept, and calibration slope improved at the longest (8 quarter) update interval (Table 1).

Formal pairwise comparisons of the difference in BS between update strategies demonstrate that all updating strategies performed better than never updating under all updating intervals (Additional file 1: Table 2), although the closed testing procedure and always refit/revision were not statistically significantly different from never updating when the update interval equaled one quarter. As the update interval increased, the performance of the closed testing procedure and always refit/revision aligned more closely with that of the recalibration strategies (Fig. 2A and Table 1), such that by the longest update interval, the differences in BS between the former two strategies and the latter were not statistically significant. When examined by quarter, all performance metrics exhibited more variability over time under the closed testing procedure and always refit/revision strategies than under the recalibration strategies (Additional file 1: Figs. 3-6).

### Varying the sliding window

Increasing the length of the sliding window had little impact on the performance of the recalibration strategies (Fig. 2B and Table 2). That is, the recalibration strategies maintain consistent performance as more old data accumulate. In contrast, the BS, AUC, H-L, calibration intercept, and calibration slope of the closed testing procedure and always refit/revision strategies were more sensitive to the sliding window length, with most metrics continuing to improve as the length of sliding window increased (i.e., as more old data were included).

Formal pairwise comparisons of the difference in BS between update strategies demonstrate that all updating strategies perform better than never updating under all sliding windows (Additional file 1: Table 3), although the closed testing procedure and always refit/revision were not statistically significantly different from never updating when only new data were used. Moreover, the always recalibrate intercept strategy exhibited comparable or better performance than both the closed testing procedure and the always recalibrate intercept and slope strategy, regardless of the sliding window length (Table 2 and Additional file 1: Table 3). As the sliding window increased, the performance of the closed testing procedure and always refit/revision strategies became more similar to—but never surpassed—that of the recalibration strategies (Table 2 and Additional file 1: Table 3).

### Number of updates

By design, the number of updates performed under the always recalibrate and always refit/revision strategies were fully determined by the length of the update interval (i.e., update intervals of 1, 2, 4, and 8 quarters corresponded to 24, 12, 6, and 3 updates, respectively). Conversely, the number of updates performed under the closed testing procedure depends on whether this approach favors updating the model over retaining the current model, with the length of the update interval defining the maximum number of updates allowed under this strategy. Tables 1 and 2 demonstrate that the closed testing procedure resulted in fewer updates relative to all other update strategies, regardless of the length of the update interval or sliding window. As the update interval and sliding windows increased, the number of updates performed under the closed testing procedure decreased.

Similarly, the number of parameters re-estimated at each update depends on the update strategy chosen, with always recalibrate intercept and always recalibrate intercept and slope re-estimating the fewest number of parameters (i.e., 1 and 2, respectively) and always refit re-estimating the most number of parameters (i.e., 15) (Table 3). The number of parameters re-estimated under the closed testing procedure falls between those two extremes (Table 3), although the actual quantity depends on the number of times the closed testing procedure selects each candidate update model (i.e., no update, recalibrate intercept, recalibrate intercept and slope, or refit). For the quarterly updates and the shortest sliding window, the closed testing procedure tended to favor no updating followed by refitting and then recalibration (Tables 4 and 5). With longer updating intervals and sliding windows, the closed testing procedure still favored no updating over the others.

**Table 3:** Number of parameters re-estimated for each update under each update strategy. For the closed testing procedure, the total number of parameters re-estimated equals the sum of the number of parameters re-estimated for each candidate update model; the actual number of parameters that change depend on which particular candidate update model is selected

Update strategy	Number of parameters re-estimated
**Never update**	0
**Closed testing procedure**	7.2*
**Always recalibrate intercept**	1
**Always recalibrate intercept and slope**	2
**Always refit (revision)**	15

*7.2 represents the weighted average of the number of parameters re-estimated under the closed testing pProcedure when the update interval equals 1 quarter. More specifically, the number of parameters re-estimated under recalibrate intercept, recalibrate slope, and refit are 1, 2, and 15, respectively. Multiplying these numbers by the total number of times the closed testing procedure selected each candidate update model (i.e., 3, 4, and 5, respectively; see Tables 4 and 5) and dividing by the total number of updates performed (i.e., 12) yields: $\frac{\left(1\ast 3\right)+\left(2\ast 4\right)+\left(15\ast 5\right)}{12}=7.2$

**Table 4:** Number of times each candidate update model was selected by the closed testing procedure when the update interval equals 1, 2, 4, and 8 quarters and 100% new data are used

Candidate update model in closed testing procedure	Update interval			
	1 quarter	2 quarters	4 quarters	8 quarters
No update	12	7	2	1
Recalibrate intercept	3	3	3	1
Recalibrate intercept and slope	4	2	0	0
Refit	5	0	1	1

**Table 5:** Number of times each candidate update model was selected by the closed testing procedure when the sliding window equals 1, 2, 4, or 8 quarters and the update interval equals 1 quarter

Candidate update model in closed testing procedure	Sliding window			
	1 quarter new	2 quarters (1 q new + 1 q old)	4 quarters (1 q new + 3 q old)	8 quarters (1 q new + 7 q old)
No update	12	16	16	16
Recalibrate intercept	3	6	2	1
Recalibrate intercept and slope	4	2	1	1
Refit	5	0	5	4

## Discussion

This study has three important findings. First, we have shown that dynamic updating of a clinical prediction model leads to improvement in overall model performance relative to not updating the model (as is done in typical practice), regardless of which update strategy is used. Second, recalibration approaches exhibited better model performance than the other methods at the most frequent updating intervals when using the most recent data, and demonstrated little change in performance across different updating intervals or sliding windows. Third, the closed testing procedure and always refit/revision strategies never performed better than the recalibration strategies and were sensitive to the amount of data used, often requiring substantially more data to yield discrimination and calibration metrics similar to those of the recalibration strategies. This last finding is likely due to the fact that the closed testing procedure and always refit/revision strategies require the re-estimation of more parameters, thus necessitating a larger sample size.

In the dynamic updating setting, the recalibration strategies had better performance characteristics than the other update strategies. This result is consistent with several other studies which compared the recalibrate intercept, recalibrate intercept and slope, and refit/revision strategies (among others) in a variety of clinical settings (e.g., acute myocardial infarction [3]; prostate cancer, traumatic brain injury, and fever [5]; and cardiac surgery [6]). However, the former two studies [3, 5] only evaluated these strategies in the single-time updating setting, and the latter [6] only compared strategies after model updating stopped. In contrast, our study reveals that model recalibration can maintain good performance in the dynamic updating setting.

An additional benefit of recalibration strategies is that only predicted and observed outcome data are needed to re-calibrate to the model. The other updating strategies explored here require individual (patient-level) data for all variables in the model, which can raise privacy and logistical concerns when sharing data across multiple institutions. However, the number of updates performed under the recalibration and refit strategies is fully determined by the length of the update interval, and was always higher than the number of updates performed under the closed testing procedure. This result is worth considering in scenarios where too many updates might be deemed undesirable (e.g., in clinical applications where the model must be approved by regulatory bodies and rolled-out to clinics in a manner which ensures that it is applied to patients in a fair and equitable manner) or infeasible (e.g., data are not processed quickly enough for analyses).

Our results suggest that there may be a tradeoff between the amount of available data and the frequency of updating. Recalibration methods may be less sensitive to smaller amounts of data but more sensitive to the use of older data. In contrast, when the amount of available data is small (e.g., quarterly update), the closed testing procedure may lack sufficient power to detect a significant difference between any of the candidate update models and the original (baseline) model. Consequently, we fail to reject the baseline model and incorrectly declare that no model updating is necessary. Even if the closed testing procedure correctly rejects the baseline model in favor of one of the other candidate update models, having limited data available can impact the accuracy of the updated model, with the refit/revised model being especially susceptible to instability and/or overfitting (see further discussion below). This last result is concerning, because in several of the situations examined here—though not all—the closed testing procedure favored the refit/revised model, consistent with Van Calster et al. [9] and Davis et al. [17]. To ameliorate such concerns, current literature recommends using “sufficiently large data sets” [4] or applying “shrinkage” to model coefficients [18].

In the case of the always refit/revision strategy, when the amount of available data is too small, there may be insufficient information to estimate certain coefficients, due to predictors with no variability between samples (e.g., in seven of the 24 post-baseline quarters, no patients had creatinine increase ≥ 150%). Thus, models fitted under the always refit/revision strategy may be more unstable (i.e., have higher variability) and have worse prediction performance over time compared to the recalibration strategies. Our finding that the recalibration strategies consistently yielded better performance in the dynamic updating setting agrees with Van Calster et al.’s recommendations to target the development of parsimonious models with “moderate calibration” rather than overly complex models with “strong calibration,” as moderate calibration is sufficient for decision-making purposes, while strong calibration can lead to overfitting [4].

Although increasing the length of the update interval and/or sliding window generally led to improved performance metrics for all update strategies, H-L actually became worse under the longest update interval (i.e., 8 quarters) and the longest sliding window (i.e., 1 quarter new + 7 quarters old data). Thus, postponing updates for too long or relying on too much old data yields inaccurate model calibration, consistent with “calibration drift” [19]. Davis et al. propose “dynamic calibration curves” to detect the extent of calibration drift and inform stakeholders when models should be updated; their method also provides a “candidate update sample” which can be used to update the model [19]. Such an approach could potentially serve to identify an upper limit to the length of the update interval and/or sliding window.

Our results can have substantial implications for implementing these strategies in practice. For example, the choice of update interval and sliding window will depend on the amount of data available and how fast they accrue. If the event rate is low or data accrue slowly, one may not be able to update frequently or use only more recent data to update, particularly for the closed testing and always refit strategies because larger sample sizes would be required to identify changes and ensure the stability of model performance. Choosing a reasonable update interval and sliding window should also incorporate substantive knowledge of the clinical application. Shorter update intervals and sliding windows may be more responsive to sudden (acute) changes in the underlying clinical population, whereas longer update intervals and sliding windows may be better equipped to handle gradual changes.

Strengths of this empiric analysis include the comparison of several different types of updating strategies in the dynamic updating setting. We also examined the sensitivity of these update strategies to different update intervals and sliding windows. Our analysis focused on the prediction of 1-year post-lung transplant survival, which is used to allocate donor organs to lung transplant candidates [13], but is not updated frequently in practice [14].

Limitations of this study include the following: First, while we considered a broad range of update intervals, it may make sense to consider different update intervals in different clinical contexts, depending on the sample size, event rate, and timeframe of analysis. Second, we did not have enough data to examine longer sliding windows at longer update intervals. However, we did explore using 50% new + 50% old data when the update interval equaled 4 quarters, and obtained comparable results as those shown in the main text (Additional file 1: Tables 4-6 and Figs. 7-12). Third, while the always recalibrate intercept strategy performed best in this study, this result may not apply in other clinical settings where, for example, there are larger changes in model covariates together with a change in incidence (model intercept) over time. Future work should explore how these update strategies perform when covariates change over time. Fourth, when dynamic updating of models is performed across multiple sites, one must consider whether to apply the update strategies to each site individually (i.e., perform site-specific updates) or to combine the data and apply the update strategies across all sites. Fifth, while we examined the performance of several different updating strategies discussed in the literature, we did not evaluate the performance of Bayesian dynamic models. Sixth, while we compared updating strategies in new cohorts of patients over time, such an approach is inherently retrospective. Research is now needed to evaluate how these updating strategies perform prospectively, particularly for varying time frames between prediction and outcome ascertainment.

## Conclusion

Overall, our study illustrates the benefit of dynamic model updating in clinical contexts. Our study was unique in that we evaluated how model predictive performance changes over time under a variety of updating strategies applied at different update intervals and based on different sliding window lengths. Recalibration strategies with more frequent updating were superior to other strategies and least sensitive to the update interval and sliding window. However, when choosing an update strategy, one should consider how much data are available (both in terms of sample size and event rate), as well as the amount of resources (e.g., computational or financial) required to perform the update and disseminate it to stakeholders.

## Supplementary Information

**Additional file 1: Table 1.** Sample size, number of deaths, and mortality risk per quarter in the post-baseline period. **Figure 1.** Proportion of deaths versus time in the post-baseline period. **Figure 2. **Overall calibration for each update strategy when update interval equals 1 quarter and 100% new data are used. Triangles represent deciles of predicted probabilities; vertical lines at bottom of plot represent the distribution of subjects stratified by outcome status (below x-axis = alive, above x-axis = dead). **Figure 3.** Brier Score versus time when update interval equals 1 quarter and 100% new data are used. **Figure 4.** AUC vs. time when update interval equals 1 quarter and 100% new data are used. **Figure 5.** Hosmer-Lemeshow statistic vs. time when update interval equals 1 quarter and 100% new data are used. **Figure 6.** Calibration vs. time when update interval equals 1 quarter and 100% new data are used. A) calibration intercept (calibration-in-the-large); B) calibration slope. **Table 2.** Pairwise comparisons (Wilcoxon Signed Rank Test) of Brier Score (BS) at each quarter when the update interval equals 1, 2, 4, or 8 quarters and 100% new data are used for each update. Values represent the median difference (*p*-value) in BS between the strategy listed in the row header and that listed in the column header. Negative differences indicate that the row header has lower BS (performs better) than the column header; positive differences indicate that the row header has higher BS (performs worse) than the column header. **Table 3.** Pairwise comparisons (Wilcoxon Signed Rank Test) of Brier Score (BS) at each quarter when the sliding window equals 1, 2, 4, or 8 quarters and the update interval equals 1 quarter. Values represent the median difference (*p*-value) in BS between the strategy listed in the row header and that listed in the column header. Negative differences indicate that the row header has lower BS (performs better) than the column header; positive differences indicate that the row header has higher BS (performs worse) than the column header. **Table 4.** Brier Score (BS), AUC, Hosmer-Lemeshow statistic, calibration intercept and slope (from logistic calibration), and number of updates performed under each update strategy when the sliding window equals 4 or 8 quarters and the update interval equals 4 quarters. **Table 5.** Number of times each candidate update model was selected by the Closed Testing Procedure when the sliding window equals 4 or 8 quarters and the update interval equals 4 quarters. **Table 6. **Pairwise comparisons (Wilcoxon Signed Rank Test) of Brier Score (BS) at each quarter when the sliding window equals 4 or 8 quarters and the update interval equals 4 quarters. Values represent the median difference (p-value) in BS between the strategy listed in the row header and that listed in the column header. Negative differences indicate that the row header has lower BS (performs better) than the column header; positive differences indicate that the row header has higher BS (performs worse) than the column header. **Figure 7.** Boxplot of difference in Brier Score between each update strategy and never update calculated at each quarter versus sliding window length, when the update interval equals 4 quarters. Horizontal bars represent medians and interquartile ranges (IQR). Negative values reflect improvement compared never update. **Figure 8.** Overall calibration for each update strategy when update interval equals 4 quarters and 100% new data are used. Triangles represent deciles of predicted probabilities; vertical lines at bottom of plot represent the distribution of subjects stratified by outcome status (below x-axis = alive, above x-axis = dead). **Figure 9.** Brier Score versus time when update interval equals 4 quarters and 100% new data are used. **Figure 10.** AUC vs. time when update interval equals 4 quarters and 100% new data are used. **Figure 11.** Hosmer-Lemeshow statistic vs. time when update interval equals 4 quarters and 100% new data are used. **Figure 12.** Calibration vs. time when update interval equals 4 quarters and 100% new data are used. A) calibration intercept (calibration-in-the-large); B) calibration slope.

## Data Availability

This study used data from the United Network for Organ Sharing (UNOS). The authors do not have the authority to share UNOS data; researchers interested in accessing this data must submit a request to UNOS directly. All code is available upon request.

## Abbreviations

AUC: Area under receiver operating characteristic curve
LAS: Lung allocation score
UNOS: United Network for Organ Sharing

## References

[1] Steyerberg EW. Updating for a New Setting. In: Clinical Prediction Models: A Practical Approach to Development, Validation, and Updating. New York: Springer Science+Business Media, LLC; 2010.

[2] Jenkins DA, Sperrin M, Martin GP, Peek N (2018). Dynamic models to predict health outcomes: current status and methodological challenges. Diagn Progn Res.

[3] Steyerberg EW, Vergouwe Y (2014). Towards better clinical prediction models: seven steps for development and an ABCD for validation. Eur Heart J.

[4] Van Calster B (2016). A calibration hierarchy for risk models was defined: from Utopia to empirical data. J Clin Epidemiol.

[5] Vergouwe Y, Nieboer D, Oostenbrink R, Debray TPA, Murray GD, Kattan MW, Koffijberg H, Moons KGM, Steyerberg EW (2017). A closed testing procedure to select an appropriate method for updating prediction models. Stat Med.

[6] Su TL, Jaki T, Hickey GL, Buchan I, Sperrin M (2018). A review of statistical updating methods for clinical prediction models. Stat Methods Med Res.

[7] Cox D (1958). Two further applications of a model for binary regression.

[8] Miller ME, Langefeld CD, Tierney WM, Hui SL, McDonald CJ (1993). Validation of probabilistic predictions. Med Decis Making.

[9] Van Calster B (2017). Validation and updating of risk models based on multinomial logistic regression. Diagn Progn Res.

[10] Hickey GL, Grant SW, Caiado C, Kendall S, Dunning J, Poullis M, Buchan I, Bridgewater B (2013). Dynamic prediction modeling approaches for cardiac surgery. Circ Cardiovasc Qual Outcomes.

[11] Minne L, Eslami S, de Keizer N, de Jonge E, de Rooij SE, Abu-Hanna A (2012). Statistical process control for monitoring standardized mortality ratios of a classification tree model. Methods Inf Med.

[12] Davis SE (2019). Comparison of prediction model performance updating protocols: using a data-driven testing procedure to guide updating. AMIA Annu Symp Proc.

[13] Organ Procurement and Transplantation Network (OPTN) Policies, effective 1 March 2020. Available: https://optn.transplant.hrsa.gov/media/1200/optn_policies.pdf. Accessed 10 Mar 2020.

[14] Gottlieb J (2017). Lung allocation. J Thorac Dis.

[15] Egan TM, Edwards LB (2016). Effect of the lung allocation score on lung transplantation in the United States. J Heart Lung Transplant.

[16] Schneeweiss S (2010). A basic study design for expedited safety signal evaluation based on electronic healthcare data. Pharmacoepidemiol Drug Saf.

[17] Davis SE, Greevy RA, Fonnesbeck C, Lasko TA, Walsh CG, Matheny ME (2019). A nonparametric updating method to correct clinical prediction model drift. J Am Med Inform Assoc.

[18] Steyerberg EW, Borsboom GJJM, van Houwelingen HC, Eijkemans MJC, Habbema JDF (2004). Validation and updating of predictive logistic regression models: a study on sample size and shrinkage. Stat Med.

[19] Davis SE, Greevy RA, Lasko TA, Walsh CG, Matheny ME (2020). Detection of calibration drift in clinical prediction models to inform model updating. J Biomed Inform.

