# Supplementary material for: Comparison of dynamic updating strategies for clinical prediction models
Source: Diagn Progn Res. 2021 Dec 6;5:20. doi: 10.1186/s41512-021-00110-w (PMC8647501; doi:10.1186/s41512-021-00110-w)
Supplement: Supplementary file 1 — Additional file 1: Table 1. Sample size, number of deaths, and mortality risk per quarter in the post-baseline period. Figure 1. Proportion of deaths versus time in the post-baseline period. Figure 2. Overall calibration for each update strategy when update interval equals 1 quarter and 100% new data are used. Triangles represent deciles of predicted probabilities; vertical lines at bottom of plot represent the distribution of subjects stratified by outcome status (below x-axis = alive, above x-axis = dead). Figure 3. Brier Score versus time when update interval equals 1 quarter and 100% new data are used. Figure 4. AUC vs. time when update interval equals 1 quarter and 100% new data are used. Figure 5. Hosmer-Lemeshow statistic vs. time when update interval equals 1 quarter and 100% new data are used. Figure 6. Calibration vs. time when update interval equals 1 quarter and 100% new data are used. A) calibration intercept (calibration-in-the-large); B) calibration slope. Table 2. Pairwise comparisons (Wilcoxon Signed Rank Test) of Brier Score (BS) at each quarter when the update interval equals 1, 2, 4, or 8 quarters and 100% new data are used for each update. Values represent the median difference (p-value) in BS between the strategy listed in the row header and that listed in the column header. Negative differences indicate that the row header has lower BS (performs better) than the column header; positive differences indicate that the row header has higher BS (performs worse) than the column header. Table 3. Pairwise comparisons (Wilcoxon Signed Rank Test) of Brier Score (BS) at each quarter when the sliding window equals 1, 2, 4, or 8 quarters and the update interval equals 1 quarter. Values represent the median difference (p-value) in BS between the strategy listed in the row header and that listed in the column header. Negative differences indicate that the row header has lower BS (performs better) than the column header; positive differences indicate that the [file 41512_2021_110_MOESM1_ESM.docx]

**Comparison of Dynamic Updating Strategies for Clinical Prediction Models**

**APPENDIX**

Table of Contents

[Sample Size and Mortality Risk 2](#_Toc82531363)

[Methodologic Details on Updating Strategies 3](#_Toc82531364)

[Visualizing Calibration for each Update Strategy 5](#_Toc82531365)

[Assessing Performance Metric Variability Over Time 6](#_Toc82531366)

[Pairwise Comparisons of Brier Score 9](#_Toc82531367)

[Examining 50% new + 50% old Data when Update Interval Equals 4 Quarters 12](#_Toc82531368)

[R Code 17](#_Toc82531369)

[Never Update 17](#_Toc82531370)

[Refit the model every quarter 18](#_Toc82531371)

[Recalibrate the intercept & slope every quarter 19](#_Toc82531372)

[Recalibrate the intercept every quarter (“recalibration-in-the-large”) 20](#_Toc82531373)

[Update model every quarter via closed testing procedure (Vergouwe et al. 2017) 22](#_Toc82531374)

[References 26](#_Toc82531375)

#

# **Sample Size and Mortality Risk**

**Appendix Table 1. Sample size, number of deaths, and mortality risk per quarter in the post-baseline period.**

| **Quarter** | **Sample Size** | **Number of Deaths** | **Mortality Risk** |
| --- | --- | --- | --- |
| 2010 Q1 | 412 | 72 | 0.175 |
| 2010 Q2 | 403 | 47 | 0.117 |
| 2010 Q3 | 453 | 63 | 0.139 |
| 2010 Q4 | 436 | 62 | 0.142 |
| 2011 Q1 | 405 | 68 | 0.168 |
| 2011 Q2 | 451 | 79 | 0.175 |
| 2011 Q3 | 483 | 61 | 0.126 |
| 2011 Q4 | 447 | 63 | 0.141 |
| 2012 Q1 | 440 | 67 | 0.152 |
| 2012 Q2 | 425 | 51 | 0.120 |
| 2012 Q3 | 450 | 53 | 0.118 |
| 2012 Q4 | 416 | 52 | 0.125 |
| 2013 Q1 | 457 | 55 | 0.120 |
| 2013 Q2 | 478 | 62 | 0.130 |
| 2013 Q3 | 466 | 69 | 0.148 |
| 2013 Q4 | 454 | 47 | 0.104 |
| 2014 Q1 | 445 | 56 | 0.126 |
| 2014 Q2 | 457 | 69 | 0.151 |
| 2014 Q3 | 503 | 72 | 0.143 |
| 2014 Q4 | 475 | 60 | 0.126 |
| 2015 Q1 | 456 | 56 | 0.123 |
| 2015 Q2 | 497 | 62 | 0.125 |
| 2015 Q3 | 523 | 58 | 0.111 |
| 2015 Q4 | 516 | 45 | 0.087 |

**Appendix Figure 1. Proportion of deaths versus time in the post-baseline period.**

**
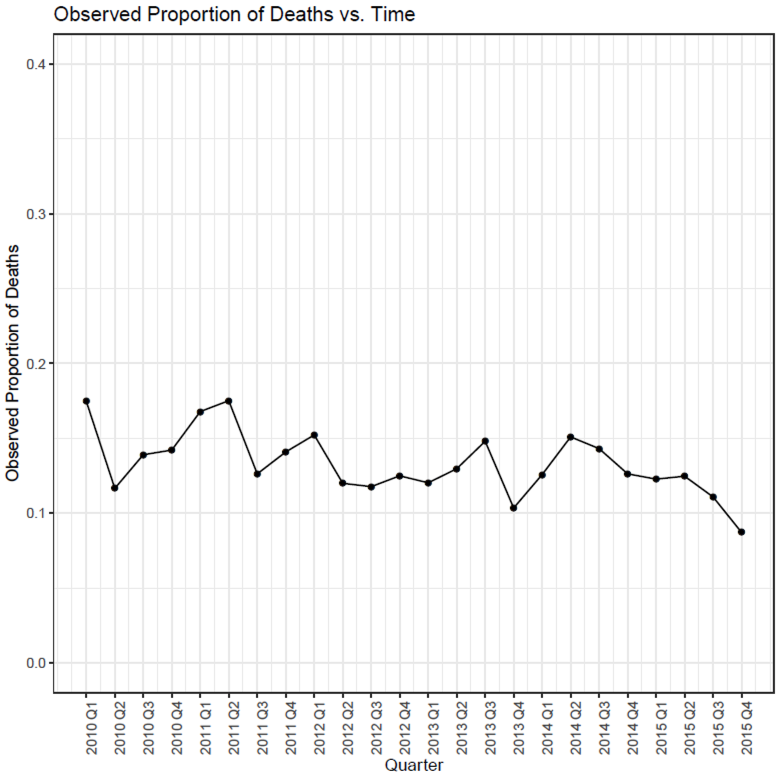
**

# **Methodologic Details on Updating Strategies**

This study examined five different update strategies: 1) never update, 2) update using the closed testing procedure proposed by Vergouwe et al. [1], 3) always recalibrate the intercept [1-6], 4) always recalibrate the intercept and slope [1-8], and 5) always refit the model (model revision [1, 6]). Here, we provide methodologic details on how each of these updating strategies is performed.

The Never Update strategy simply applies the baseline prediction model (i.e., the logistic regression model for one-year post-lung transplant mortality fit using the baseline period data and the same covariates as the 2010 post-transplant LAS model) to all subsequent quarters in the post-baseline period.

The Always Recalibrate Intercept strategy – also referred to in the literature as “recalibration in the large” [1, 4, 6] – involves updating the intercept of the prediction model at each of the times stipulated by the chosen update interval, using either all new data accrued since the previous update (in the update interval analysis) or some combination of new and old data (in the sliding window analysis). At the first update time, the intercept of the baseline prediction model is updated by fitting a logistic regression model in the “update sample” (e.g., all new data) with the intercept as a free parameter and the linear predictor from the baseline prediction model as an offset term. Using an offset term forces the slope of the recalibrated model to be the same as in the baseline model. Subsequent updates under the Always Recalibrate Intercept strategy follow the same procedure, but use the linear predictor from the most recent update of the prediction model as the offset term.

The Always Recalibrate Intercept & Slope strategy – also referred to in the literature as “recalibration” [1, 5-8] – involves updating the intercept and slope of the prediction model at each of the times stipulated by the chosen update interval, using either all new data accrued since the previous update (in the update interval analysis) or some combination of new and old data (in the sliding window analysis). At the first update time, the intercept and slope of the baseline prediction model are updated by fitting a logistic regression model in the update sample with the linear predictor from the baseline model as the only covariate. The slope of the updated model then serves as a scaling factor which rescales the baseline slope. Subsequent updates under the Always Recalibrate Intercept & Slope strategy follow the same procedure, but use the linear predictor from the most recent update of the prediction model as the covariate.

The Always Refit strategy – also referred to in the literature as “model revision” [1, 6] – involves re-estimating all coefficients of the prediction model at each of the times stipulated by the chosen update interval, using either all new data accrued since the previous update (in the update interval analysis) or some combination of new and old data (in the sliding window analysis). At each update time, the coefficients of the prediction model are updated by fitting a new logistic regression model in the update sample.

The Closed Testing Procedure proposed by Vergouwe et al. [1] involves testing for a change in the intercept and/or slope of the model via a series of sequential likelihood ratio tests (LRT) and then choosing one of the remaining four candidate update models – never update, recalibrate intercept, recalibrate intercept and slope, and refit (revise) all model coefficients [1]. The three LRT tests considered in the Closed Testing Procedure are: 1) compare the refit/revised model to the baseline model, 2) compare the refit/revised model to the recalibrated intercept model, and 3) compare the refit/revised model to the recalibrated intercept & slope model. These LRT tests are performed sequentially at a significance level of $\alpha=0.05$ using $p+1$, $p$, and $p-1$ degrees of freedom, respectively, where $p$ represents the number of coefficients in the logistic regression model (excluding the intercept). If the first LRT test is not statistically significant, the baseline prediction model is retained; otherwise, the second LRT test is performed. If the second LRT test is not statistically significant, the recalibrated intercept model is retained; otherwise, the third LRT is performed. If the third LRT test is not statistically significant, the recalibrated intercept & slope model is retained; otherwise, the refit/revised model is retained. In this study, the Closed Testing Procedure was performed at each of the times stipulated by the chosen update interval, regardless of which candidate update model was selected at the prior timepoint. As with the other strategies, the Closed Testing Procedure used all new data accrued since the previous update in the update interval analysis, and some combination of new and old data in the sliding window analysis.

# **Visualizing Calibration for each Update Strategy**

In the main text, we evaluated the calibration of each update strategy via the calibration intercept and slope (Tables 1 and 2). Here, plot observed versus predicted probabilities to illustrate calibration for each update strategy when the update interval equals 1 quarter and 100% new data are used.

**Appendix Figure 2. Overall calibration for each update strategy when update interval equals 1 quarter and 100% new data are used. Triangles represent deciles of predicted probabilities; vertical lines at bottom of plot represent the distribution of subjects stratified by outcome status (below x-axis = alive, above x-axis = dead).**

**
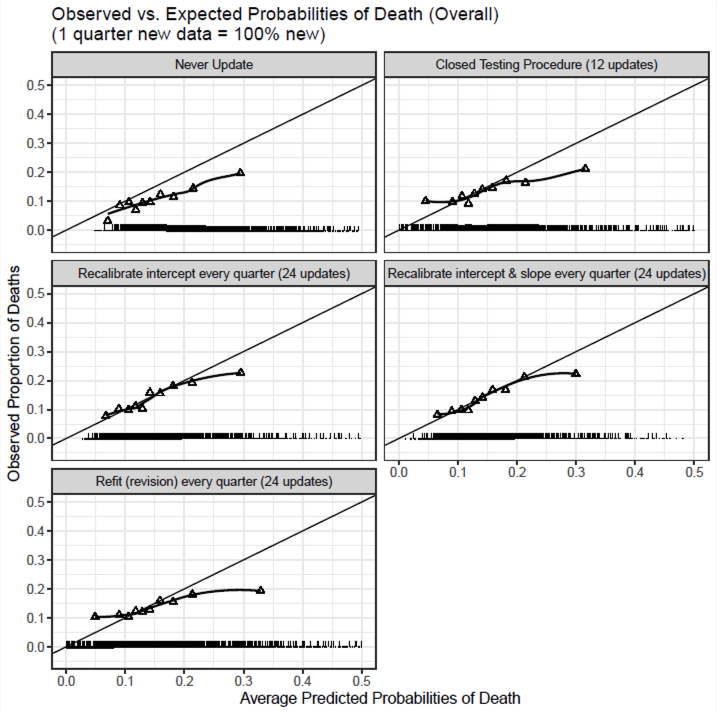
**

#

# **Assessing Performance Metric Variability Over Time**

Tables 1 and 2 in the main text display the Brier Score (BS), discrimination (AUC), and calibration (Hosmer-Lemeshow, H-L; calibration intercept; calibration slope) statistics calculated across all post-baseline quarters for each update strategy (i.e., Never Update, Closed Testing Procedure, Always Recalibrate Intercept, Always Recalibrate Intercept & Slope, and Always Refit/Revision). To assess the variability of these performance metrics, we calculated each metric by quarter, and plotted the quarterly estimates over time. Appendix Figures 3-6 display the quarterly estimates of BS, AUC, H-L, and calibration intercept and slope, respectively. When examined by quarter, all performance metrics exhibited more variability over time under the Closed Testing Procedure and Always Refit/Revision strategies than under the Recalibration strategies.

**Appendix Figure 3. Brier Score versus time when update interval equals 1 quarter and 100% new data are used.**

**
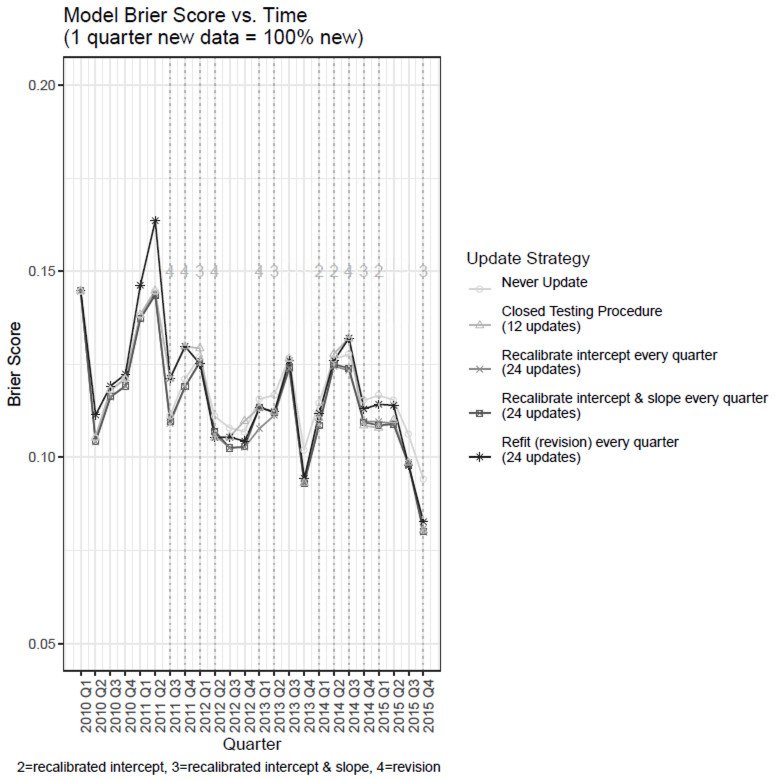
**

**Appendix Figure 4. AUC vs. time when update interval equals 1 quarter and 100% new data are used.**

**
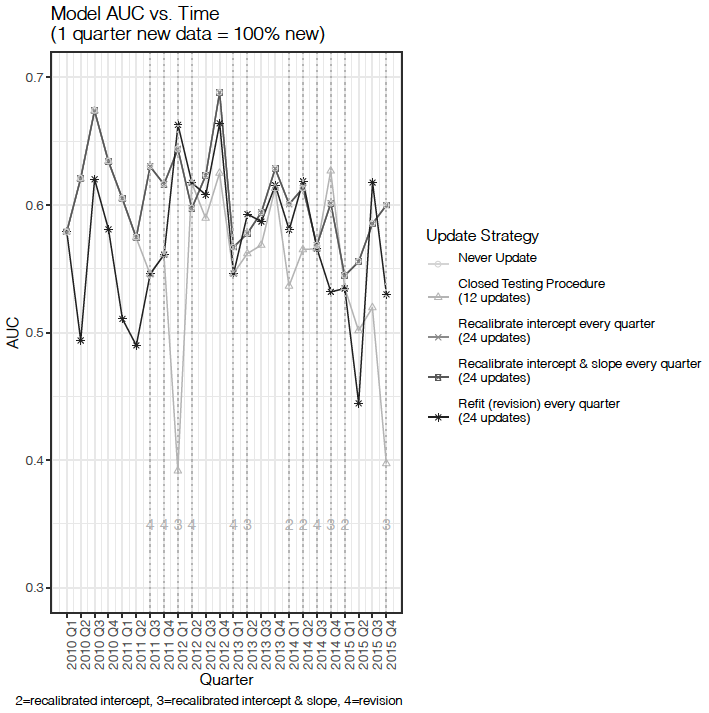
**

**Appendix Figure 5. Hosmer-Lemeshow statistic vs. time when update interval equals 1 quarter and 100% new data are used.**

**
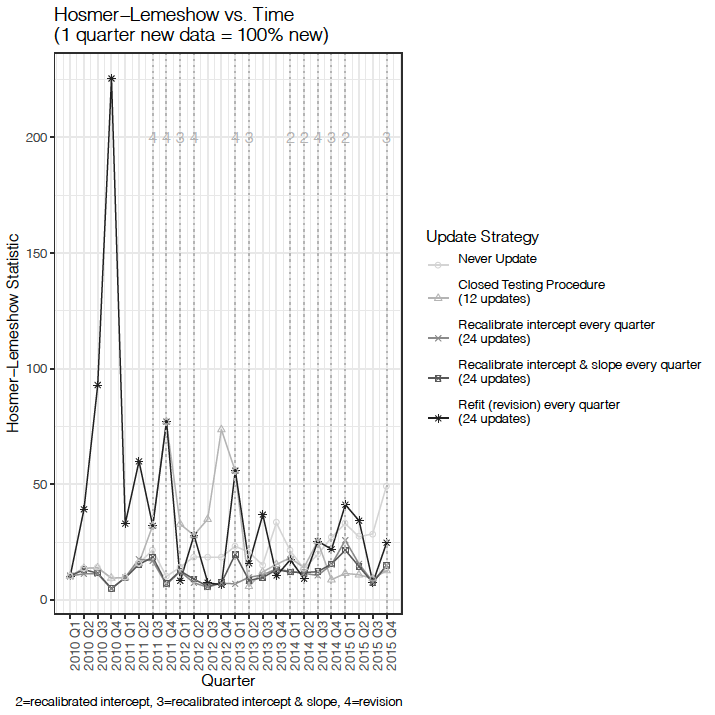
**

**Appendix Figure 6. Calibration vs. time when update interval equals 1 quarter and 100% new data are used. A) calibration intercept (calibration-in-the-large); B) calibration slope.**

**
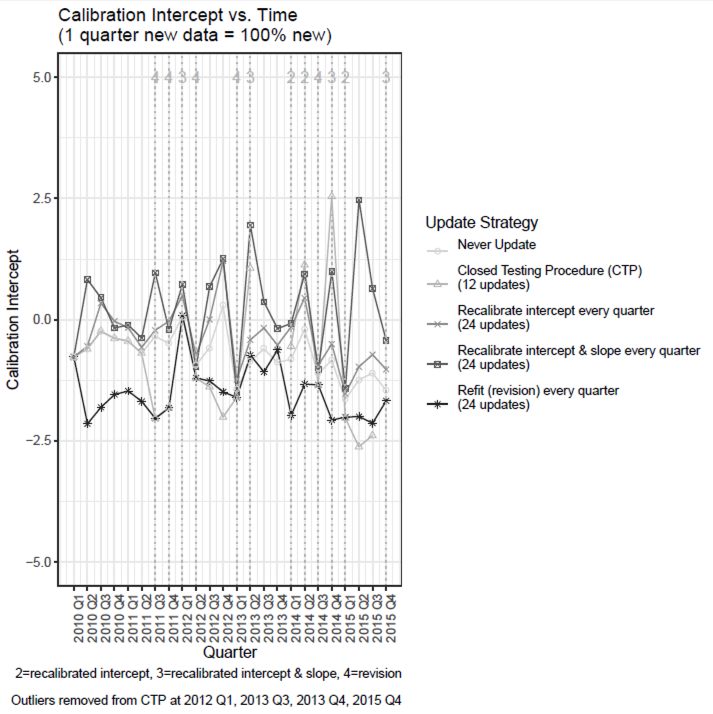
**

**A**

**
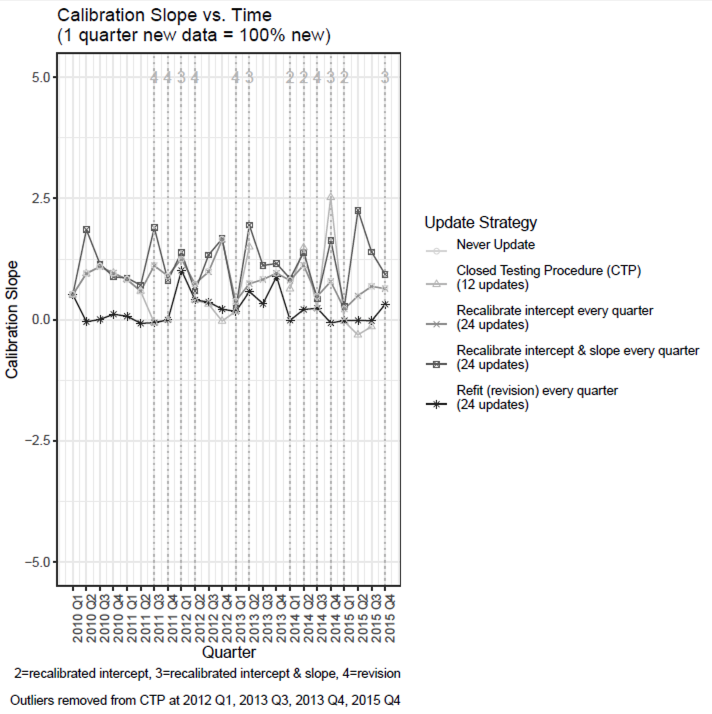
**

**B**

# **Pairwise Comparisons of Brier Score**

Figure 1 in the main text displays the Brier Score (BS) improvement for each of the update strategies (i.e., the difference in BS between each update strategy and never update at each quarter). We also computed the difference in BS between pairs of strategies at each individual quarter in the post-baseline data and performed Wilcoxon Signed Rank tests to determine which update strategy performed best.

Appendix Table 2 shows the median difference in BS between pairs of strategies, along with the corresponding p-value from the Wilcoxon Signed Rank test, when the update interval equals 1, 2, 4, and 8 quarters. These results demonstrate that all updating strategies performed better than never updating under all updating intervals, although the Closed Testing Procedure and Always Refit/Revision were not statistically significantly different from never updating when the update interval equaled one quarter.

Appendix Table 3 shows the median difference in BS between pairs of strategies, along with the corresponding p-value from the Wilcoxon Signed Rank test, when the update interval equals 1 quarter and the sliding window equals 1, 2, 4, or 8 quarters. These results demonstrate that all updating strategies perform better than never updating under all sliding windows, although the Closed Testing Procedure and Always Refit/Revision were not statistically significantly different from never updating when only new data were used.

**Appendix Table 2. Pairwise comparisons (Wilcoxon Signed Rank Test) of Brier Score (BS) at each quarter when the update interval equals 1, 2, 4, or 8 quarters and 100% new data are used for each update. Values represent the median difference (p-value) in BS between the strategy listed in the row header and that listed in the column header. Negative differences indicate that the row header has lower BS (performs better) than the column header; positive differences indicate that the row header has higher BS (performs worse) than the column header.**

| **Update Interval = 1 Quarter** | **Never Update** | **Closed Testing Procedure** | **Always Recalibrate Intercept** | **Always Recalibrate Intercept & Slope** | **Always Refit (Revision)** |
| --- | --- | --- | --- | --- | --- |
| **Never Update** |  | 0.0001 (p=0.1634) | 0.0042 (p=2.9e-05) | 0.0032 (p=2.9e-05) | 0.0016 (p=0.5531) |
| **Closed Testing Procedure** | -0.0001 (p=0.1634) |  | 0.0014 (p=0.0013) | 0.0011 (p=0.0008) | -8.34e-06 (p=0.0742) |
| **Always Recalibrate Intercept** | -0.0042 (p=2.9e-05) | -0.0014 (p=0.0013) |  | -4.56e-06 (p=0.6592) | -0.0031 (p=6.35e-05) |
| **Always Recalibrate Intercept & Slope** | -0.0032 (p=2.9e-05) | -0.0011 (p=0.0008) | 4.56e-06 (p=0.6592) |  | -0.0029 (p=0.0002) |
| **Always Refit (Revision)** | -0.0016 (p=0.5531) | 8.34e-06 (p=0.0742) | 0.0031 (p=6.35e-05) | 0.0029 (p=0.0002) |  |

| **Update Interval = 2 Quarters** | **Never Update** | **Closed Testing Procedure** | **Always Recalibrate Intercept** | **Always Recalibrate Intercept & Slope** | **Always Refit (Revision)** |
| --- | --- | --- | --- | --- | --- |
| **Never Update** |  | 0.0045 (0.0007) | 0.0044 (p=4.30e-05) | 0.0044 (p=4.95e-05) | 0.0024 (p=0.0020) |
| **Closed Testing Procedure** | -0.0045 (0.0007) |  | 0.0001 (p=0.0040) | 2.08e-05 (p=0.0186) | -0.0003 (p=0.3301) |
| **Always Recalibrate Intercept** | -0.0044 (p=4.30e-05) | -0.0001 (p=0.0040) |  | 0.0000 (p=0.8710) | -0.0010 (p=0.0212) |
| **Always Recalibrate Intercept & Slope** | -0.0044 (p=4.95e-05) | -2.08e-05 (p=0.0186) | 0.0000 (p=0.8710) |  | -0.0008 (p=0.0163) |
| **Always Refit (Revision)** | -0.0024 (p=0.0020) | 0.0003 (p=0.3301) | 0.0010 (p=0.0212) | 0.0008 (p=0.0163) |  |

| **Update Interval = 4 Quarters** | **Never Update** | **Closed Testing Procedure** | **Always Recalibrate Intercept** | **Always Recalibrate Intercept & Slope** | **Always Refit (Revision)** |
| --- | --- | --- | --- | --- | --- |
| **Never Update** |  | 0.0039 (p=9.57e-05) | 0.0040 (p=9.57e-05) | 0.0041 (p=9.57e-05) | 0.0031 (p=0.0003) |
| **Closed Testing Procedure** | -0.0039 (p=9.57e-05) |  | 0.0000 (p=0.6833) | 0.0000 (p=0.9851) | 0.0000 (p=0.1550) |
| **Always Recalibrate Intercept** | -0.0040 (p=9.57e-05) | 0.0000 (p=0.6833) |  | 1.30e-05 (p=0.5379) | -2.80e-05 (p=0.1506) |
| **Always Recalibrate Intercept & Slope** | -0.0041 (p=9.57e-05) | 0.0000 (p=0.9851) | -1.30e-05 (p=0.5379) |  | -0.0003 (p=0.1403) |
| **Always Refit (Revision)** | -0.0031 (p=0.0003) | 0.0000 (p=0.1550) | 2.80e-05 (p=0.1506) | 0.0003 (p=0.1403) |  |

| **Update Interval = 8 Quarters** | **Never Update** | **Closed Testing Procedure** | **Always Recalibrate Intercept** | **Always Recalibrate Intercept & Slope** | **Always Refit (Revision)** |
| --- | --- | --- | --- | --- | --- |
| **Never Update** |  | 0.0041 (p=0.0006) | 0.0041 (p=0.0005) | 0.0039 (p=0.0005) | 0.0032 (p=0.0006) |
| **Closed Testing Procedure** | -0.0041 (p=0.0006) |  | 0.0000 (p=0.5286) | 8.76e-05 (p=0.0411) | 0.0000 (p=0.7263) |
| **Always Recalibrate Intercept** | -0.0041 (p=0.0005) | 0.0000 (p=0.5286) |  | 9.37e-05 (p=0.0411) | 0.0000 (p=0.3655) |
| **Always Recalibrate Intercept & Slope** | -0.0039 (p=0.0005) | -8.76e-05 (p=0.0411) | -9.37e-05 (p=0.0411) |  | -0.0001 (p=0.0744) |
| **Always Refit (Revision)** | -0.0032 (p=0.0006) | 0.0000 (p=0.7263) | 0.0000 (p=0.3655) | 0.0001 (p=0.0744) |  |

**Appendix Table 3. Pairwise comparisons (Wilcoxon Signed Rank Test) of Brier Score (BS) at each quarter when the sliding window equals 1, 2, 4, or 8 quarters and the update interval equals 1 quarter. Values represent the median difference (p-value) in BS between the strategy listed in the row header and that listed in the column header. Negative differences indicate that the row header has lower BS (performs better) than the column header; positive differences indicate that the row header has higher BS (performs worse) than the column header.**

| **1 quarter new (100% new)** | **Never Update** | **Closed Testing Procedure** | **Always Recalibrate Intercept** | **Always Recalibrate Intercept & Slope** | **Always Refit (Revision)** |
| --- | --- | --- | --- | --- | --- |
| **Never Update** |  | 0.0001 (p=0.1634) | 0.0042 (p=2.9e-05) | 0.0032 (p=2.9e-05) | 0.0016 (p=0.5531) |
| **Closed Testing Procedure** | -0.0001 (p=0.1634) |  | 0.0014 (p=0.0013) | 0.0011 (p=0.0008) | -8.34e-06 (p=0.0742) |
| **Always Recalibrate Intercept** | -0.0042 (p=2.9e-05) | -0.0014 (p=0.0013) |  | -4.56e-06 (p=0.6592) | -0.0031 (p=6.35e-05) |
| **Always Recalibrate Intercept & Slope** | -0.0032 (p=2.9e-05) | -0.0011 (p=0.0008) | 4.56e-06 (p=0.6592) |  | -0.0029 (p=0.0002) |
| **Always Refit (Revision)** | -0.0016 (p=0.5531) | 8.34e-06 (p=0.0742) | 0.0031 (p=6.35e-05) | 0.0029 (p=0.0002) |  |

| **1 quarter new + 1 quarter old (50% new, 50% old)** | **Never Update** | **Closed Testing Procedure** | **Always Recalibrate Intercept** | **Always Recalibrate Intercept & Slope** | **Always Refit (Revision)** |
| --- | --- | --- | --- | --- | --- |
| **Never Update** |  | 0.0038 (p=8.58e-05) | 0.0043 (p=2.89e-05) | 0.0040 (p=2.89e-05) | 0.0021 (p=0.0030) |
| **Closed Testing Procedure** | -0.0038 (p=8.58e-05) |  | 0.0000 (p=0.0967) | 0.0001 (p=0.0194) | -0.0004 (p=0.0703) |
| **Always Recalibrate Intercept** | -0.0043 (p=2.89e-05) | 0.0000 (p=0.0967) |  | 1.94e-05 (p=0.5945) | -0.0012 (p=0.0049) |
| **Always Recalibrate Intercept & Slope** | -0.0040 (p=2.89e-05) | -0.0001 (p=0.0194) | -1.94e-05 (p=0.5945) |  | -0.0010 (p=0.0045) |
| **Always Refit (Revision)** | -0.0021 (p=0.0030) | 0.0004 (p=0.0703) | 0.0012 (p=0.0049) | 0.0010 (p=0.0045) |  |

| **1 quarter new + 3 quarters old (25% new, 75% old)** | **Never Update** | **Closed Testing Procedure** | **Always Recalibrate Intercept** | **Always Recalibrate Intercept & Slope** | **Always Refit (Revision)** |
| --- | --- | --- | --- | --- | --- |
| **Never Update** |  | 0.0029 (p=7.21e-05) | 0.0044 (p=2.89e-05) | 0.0044 (p=2.89e-05) | 0.0034 (p=9.29e-05) |
| **Closed Testing Procedure** | -0.0029 (p=7.21e-05) |  | 0.0004 (p=0.1573) | 0.0002 (p=0.0803) | 0.0000 (p=0.6012) |
| **Always Recalibrate Intercept** | -0.0044 (p=2.89e-05) | -0.0004 (p=0.1573) |  | 8.06e-05 (p=0.0974) | -7.86e-05 (p=0.2416) |
| **Always Recalibrate Intercept & Slope** | -0.0044 (p=2.89e-05) | -0.0002 (p=0.0803) | -8.06e-05 (p=0.0974) |  | -0.0002 (p=0.0857) |
| **Always Refit (Revision)** | -0.0034 (p=9.29e-05) | 0.0000 (p=0.6012) | 7.86e-05 (p=0.2416) | 0.0002 (p=0.0857) |  |

| **1 quarter new + 7 quarters old (12.5% new, 87.5% old)** | **Never Update** | **Closed Testing Procedure** | **Always Recalibrate Intercept** | **Always Recalibrate Intercept & Slope** | **Always Refit (Revision)** |
| --- | --- | --- | --- | --- | --- |
| **Never Update** |  | 0.0033 (p=4.90e-05) | 0.0043 (p=2.89e-05) | 0.0042 (p=2.89e-05) | 0.0035 (p=3.30e-05) |
| **Closed Testing Procedure** | -0.0033 (p=4.90e-05) |  | 0.0004 (p=0.0752) | 0.0005 (p=0.0078) | 0.0004 (p=0.0119) |
| **Always Recalibrate Intercept** | -0.0043 (p=2.89e-05) | -0.0004 (p=0.0752) |  | 0.0002 (p=0.0170) | -0.0001 (p=0.9394) |
| **Always Recalibrate Intercept & Slope** | -0.0042 (p=2.89e-05) | -0.0005 (p=0.0078) | -0.0002 (p=0.0170) |  | 0.0002 (p=0.3380) |
| **Always Refit (Revision)** | -0.0035 (p=3.30e-05) | -0.0004 (p=0.0119) | 0.0001 (p=0.9394) | 0.0002 (p=0.3380) |  |

# **Examining 50% new + 50% old Data when Update Interval Equals 4 Quarters**

In the main text, we considered a variety of sliding window lengths when the update interval equaled 1 quarter. While we did not have enough data to examine longer sliding windows at longer update intervals, we did explore using 50% new + 50% old data when the update interval equaled 4 quarters. The results below are comparable to the ones shown in the main text.

**Appendix Table 4. Brier Score (BS), AUC, Hosmer-Lemeshow statistic, calibration intercept and slope (from logistic calibration), and number of updates performed under each update strategy when the sliding window equals 4 or 8 quarters and the update interval equals 4 quarters.**

| **Sliding Window** | **Metric** | **4 Quarters New** | **8 Quarters**  **(4 q new + 4 q old)** |
| --- | --- | --- | --- |
| **Never Update** | BS | 0.118 | 0.118 |
|  | AUC | 0.603 | 0.603 |
|  | H-L | 290.5 | 290.5 |
|  | Calibration Intercept | -0.754 | -0.754 |
|  | Calibration Slope | 0.791 | 0.791 |
|  | # Updates | 0 | 0 |
| **Closed Testing Procedure** | BS | 0.114 | 0.114 |
|  | AUC | 0.598 | 0.601 |
|  | H-L | 37.80 | 54.07 |
|  | Calibration Intercept | -0.570 | -0.819 |
|  | Calibration Slope | 0.724 | 0.602 |
|  | # Updates | 4 | 4 |
| **Always Recalibrate Intercept** | BS | 0.114 | 0.114 |
|  | AUC | 0.603 | 0.605 |
|  | H-L | 39.21 | 39.31 |
|  | Calibration Intercept | -0.525 | -0.519 |
|  | Calibration Slope | 0.760 | 0.774 |
|  | # Updates | 6 | 6 |
| **Always Recalibrate Intercept & Slope** | BS | 0.114 | 0.113 |
|  | AUC | 0.602 | 0.604 |
|  | H-L | 35.41 | 33.68 |
|  | Calibration Intercept | -0.443 | -0.389 |
|  | Calibration Slope | 0.809 | 0.851 |
|  | # Updates | 6 | 6 |
| **Always Refit (Revision)** | BS | 0.114 | 0.114 |
|  | AUC | 0.592 | 0.601 |
|  | H-L | 56.25 | 41.94 |
|  | Calibration Intercept | -0.972 | -0.817 |
|  | Calibration Slope | 0.499 | 0.598 |
|  | # Updates | 6 | 6 |

**Appendix Table 5. Number of times each candidate update model was selected by the Closed Testing Procedure when the sliding window equals 4 or 8 quarters and the update interval equals 4 quarters.**

| **Candidate Update Model**  **in Closed Testing Procedure** | **Sliding Window** | | |
| --- | --- | --- | --- |
|  | **4 Quarters New** | **8 Quarters**  **(4 q new + 4 q old)** |  |
| Recalibrate Intercept | 3 | 1 |  |
| Recalibrate Intercept & Slope | 0 | 1 |  |
| Refit | 1 | 2 |  |
| Total number of updates performed  Maximum possible number of updates | 4  6 | 4  6 |  |

**Appendix Table 6. Pairwise comparisons (Wilcoxon Signed Rank Test) of Brier Score (BS) at each quarter when the sliding window equals 4 or 8 quarters and the update interval equals 4 quarters. Values represent the median difference (p-value) in BS between the strategy listed in the row header and that listed in the column header. Negative differences indicate that the row header has lower BS (performs better) than the column header; positive differences indicate that the row header has higher BS (performs worse) than the column header.**

| **4 quarters new (100% new)** | **Never Update** | **Closed Testing Procedure** | **Always Recalibrate Intercept** | **Always Recalibrate Intercept & Slope** | **Always Refit (Revision)** |
| --- | --- | --- | --- | --- | --- |
| **Never Update** |  | 0.0039 (p=9.57e-05) | 0.0040 (p=9.57e-05) | 0.0041 (p=9.57e-05) | 0.0031 (p=0.0003) |
| **Closed Testing Procedure** | -0.0039 (p=9.57e-05) |  | 0.0000 (p=0.6833) | 0.0000 (p=0.9851) | 0.0000 (p=0.1550) |
| **Always Recalibrate Intercept** | -0.0040 (p=9.57e-05) | 0.0000 (p=0.6833) |  | 1.30e-05 (p=0.5379) | -2.80e-05 (p=0.1506) |
| **Always Recalibrate Intercept & Slope** | -0.0041 (p=9.57e-05) | 0.0000 (p=0.9851) | -1.30e-05 (p=0.5379) |  | -0.0003 (p=0.1403) |
| **Always Refit (Revision)** | -0.0031 (p=0.0003) | 0.0000 (p=0.1550) | 2.80e-05 (p=0.1506) | 0.0003 (p=0.1403) |  |

| **4 quarters new + 4 quarters old (50% new, 50% old)** | **Never Update** | **Closed Testing Procedure** | **Always Recalibrate Intercept** | **Always Recalibrate Intercept & Slope** | **Always Refit (Revision)** |
| --- | --- | --- | --- | --- | --- |
| **Never Update** |  | 0.0035 (p=2.36e-04) | 0.0041 (p=9.57e-05) | 0.0039 (p=9.57e-05) | 0.0035 (p=1.51e-04) |
| **Closed Testing Procedure** | -0.0035 (p=2.36e-04) |  | 0.0000 (p=0.5521) | 1.28e-05 (p=0.0525) | 0.0000 (p=0.3268) |
| **Always Recalibrate Intercept** | -0.0041 (p=9.57e-05) | 0.0000 (p=0.5521) |  | 1.34e-04 (p=0.0239) | 0.0000 (p=0.7510) |
| **Always Recalibrate Intercept & Slope** | -0.0039 (p=9.57e-05) | -1.28e-05 (p=0.0525) | -1.34e-04 (p=0.0239) |  | 0.0000 (p=0.2396) |
| **Always Refit (Revision)** | -0.0035 (p=1.51e-04) | 0.0000 (p=0.3268) | 0.0000 (p=0.7510) | 0.0000 (p=0.2396) |  |

**Appendix Figure 7. Boxplot of difference in Brier Score between each update strategy and never update calculated at each quarter versus sliding window length, when the update interval equals 4 quarters. Horizontal bars represent medians and interquartile ranges (IQR). Negative values reflect improvement compared never update.**


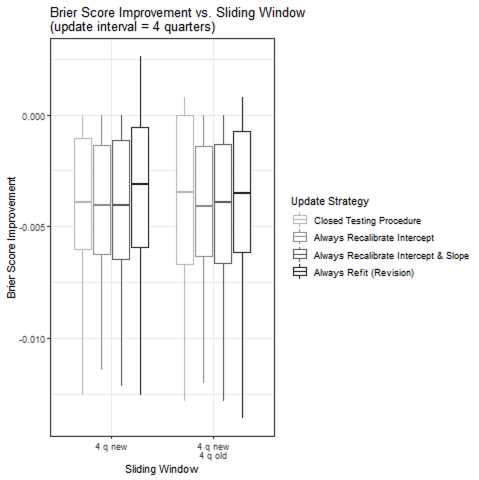


**Appendix Figure 8. Overall calibration for each update strategy when update interval equals 4 quarters and 100% new data are used. Triangles represent deciles of predicted probabilities; vertical lines at bottom of plot represent the distribution of subjects stratified by outcome status (below x-axis = alive, above x-axis = dead).**

**
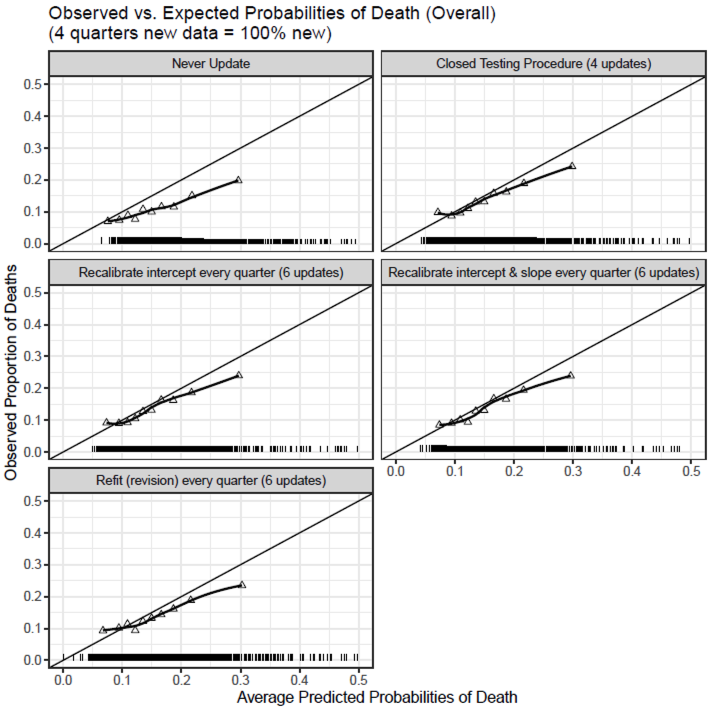
**

**Appendix Figure 9. Brier Score versus time when update interval equals 4 quarters and 100% new data are used.**

**
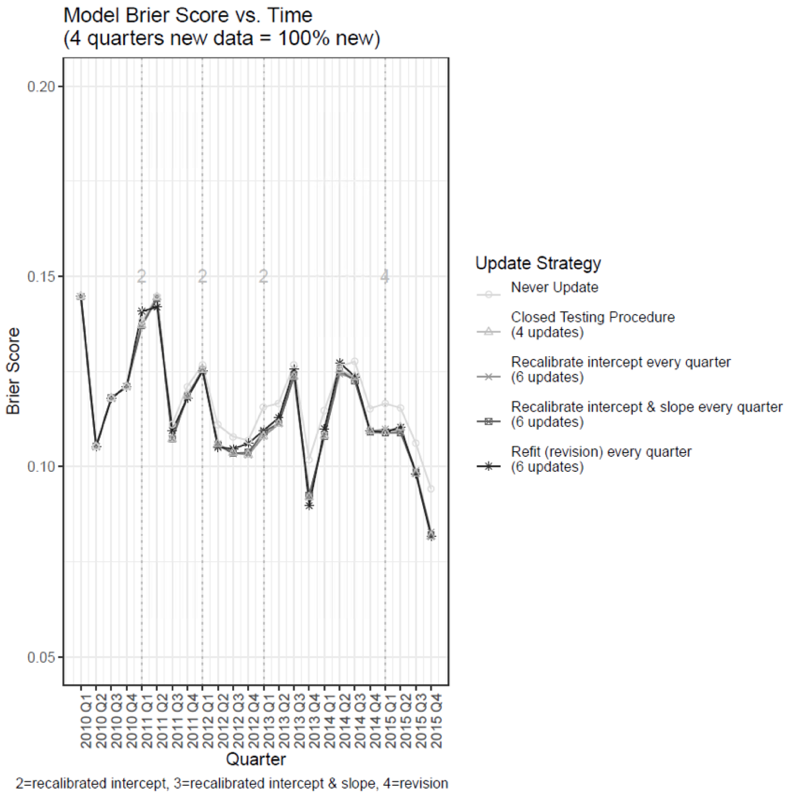
**

**Appendix Figure 10. AUC vs. time when update interval equals 4 quarters and 100% new data are used.**

**
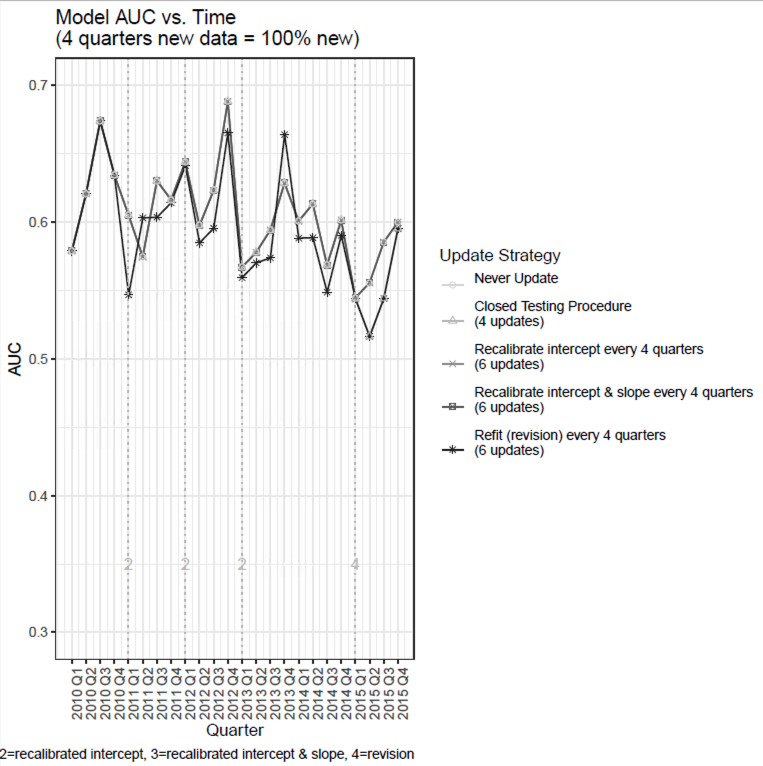
**

**Appendix Figure 11. Hosmer-Lemeshow statistic vs. time when update interval equals 4 quarters and 100% new data are used.**

**
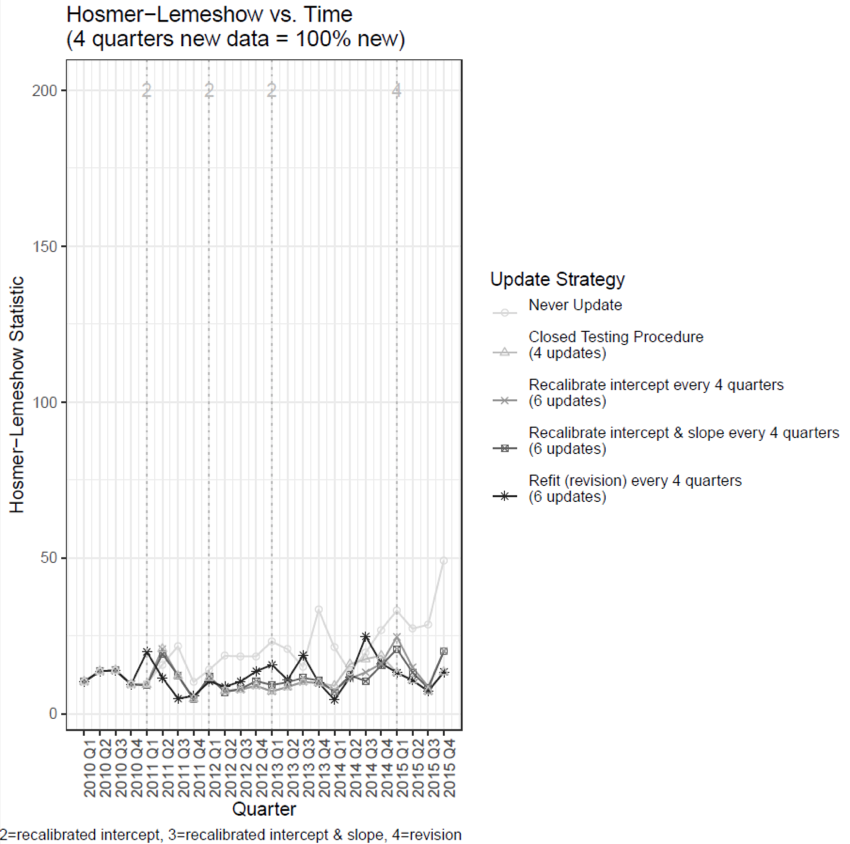
**

**Appendix Figure 12. Calibration vs. time when update interval equals 4 quarters and 100% new data are used. A) calibration intercept (calibration-in-the-large); B) calibration slope.**

**
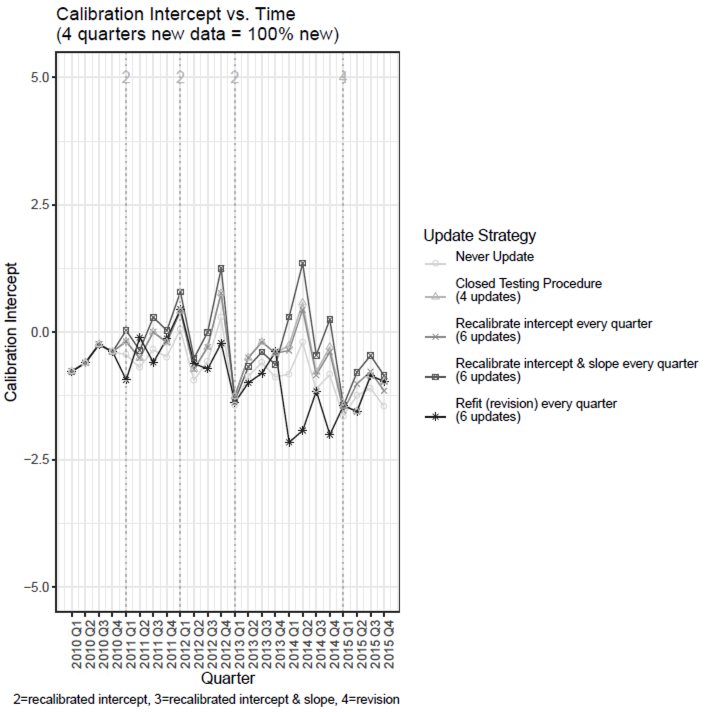
**

**A**

**
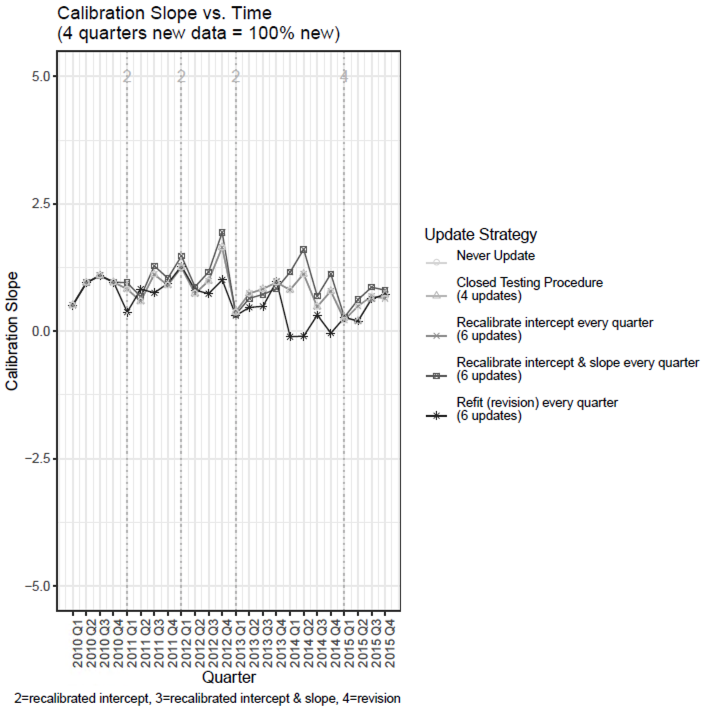
**

**B**

# **R Code**

Below we include the R code used to implement the five different update strategies described in the main text (i.e., never update, update using the closed testing procedure proposed by Vergouwe et al. [1], always recalibrate the intercept [1-6], always recalibrate the intercept and slope [1-8], and always refit the model [1, 6]) when the update interval equaled one quarter and 100% new data were used (i.e., the reference case). Similar code was used to conduct the remaining update interval and sliding window analyses.

### Never Update

Here, we fit a baseline prediction model using the same data that was used to derive the 2010 post-transplant LAS model. We then assess the performance of this model each quarter between 2010 and 2015.

############################
# Strategy A: Never Update #
############################

# Fit baseline prediction model using the UNOSpre dataframe
 baseline_int_slope_model <- glm(pstatus2 ~ agespline + #age
 ci_bin + calc_vent_use_bin + end_creat + creatinc2_flag +
 dx_group + func_stat_trr_bin + calc_o2 + calc_o2*dx_group + # o2 differs for groups B, C, D
 walkspline, #calc_six_min_walk,
 data=UNOSpre_sort,
 family="binomial")


## Perform the remaining steps in a loop

for (i in 1:length(quarter_post)){ # Exclude baseline period

 # Store summary of baseline prediction model in master list for future reference
 StrategyA_lm_master[[i]] <- summary(baseline_int_slope_model)


 # Apply baseline model to each subsequent quarter (but exclude baseline quarters)
 baseline_int_slope_predprob <- predict(baseline_int_slope_model,
 filter(UNOSpost_sort, quarter==i),
 type="response")

 # Create data frame with observed and predicted probabilities
 StrategyA_predmat <- as.data.frame(cbind(as.numeric(as.character(filter(UNOSpost_sort, quarter==i)$quarter)),
 as.numeric(as.character(filter(UNOSpost_sort, quarter==i)$pstatus2)),
 baseline_int_slope_predprob))
 names(StrategyA_predmat) <- c("quarter", "pstatus2", "predprob")

 # Store this data in the "master" list for future use
 StrategyA_predmat_master[[i]] <- StrategyA_predmat


 # Compute C-statistic in quarter i using Harrell's rms package. See:
 # https://stats.stackexchange.com/questions/145566/how-to-calculate-area-under-the-curve-auc-or-the-c-statistic-by-hand

 AUC_StrategyA[i] <- as.numeric(rcorr.cens(StrategyA_predmat$predprob, StrategyA_predmat$pstatus2))[1]


 # Compute the Mean Absolute Error (MAE) in quarter i
 MAE_StrategyA[i] <- mean(abs(StrategyA_predmat$predprob-StrategyA_predmat$pstatus2), na.rm=TRUE)


 # Compute Brier Score in quarter i
 Brier_StrategyA[i] <- mean((StrategyA_predmat$predprob-StrategyA_predmat$pstatus2)^2, na.rm=TRUE)


}

# Convert the predmat list into a dataframe
StrategyA_predmat_allupdates <- do.call(rbind.data.frame, StrategyA_predmat_master)

# Rename as StrategyA_predmat so we don't have to update future lines of code
StrategyA_predmat <- StrategyA_predmat_allupdates

###

### Refit the model every quarter

Here, we refit the model every quarter using the prior quarter of data.

#####################################################################
# Strategy D_1: Refit every quarter using the prior quarter of data #
#####################################################################

## Perform the remaining steps in a loop

for (i in 2:length(quarter_post)){ # Exclude baseline period

 # Refit the model using the prior quarter of data
 # Use numeric version of binary variables to avoid error that occurs if only one level of the factor is present in a given quarter
 update_int_slope_model <- glm(pstatus2 ~ agespline + #age
 as.numeric(as.character(ci_bin)) +
 as.numeric(as.character(calc_vent_use_bin)) +
 end_creat +
 as.numeric(as.character(creatinc2_flag)) +
 dx_group +
 as.numeric(as.character(func_stat_trr_bin)) +
 calc_o2 +
 calc_o2*dx_group + # o2 differs for groups B, C, D
 walkspline, #calc_six_min_walk,
 data=filter(UNOSpost_sort, quarter==(i-1)),
 family="binomial")

 # Store summary of refitted model at each quarter in master list for future reference
 StrategyD_1_lm_master[[i]] <- summary(update_int_slope_model)

 # Obtain predicted probabilities for the next quarter
 update_int_slope_predprob <- predict(update_int_slope_model,
 filter(UNOSpost_sort, quarter==i),
 type="response")

 # Create data frame with observed and predicted probabilities
 StrategyD_1_predmat <- as.data.frame(cbind(as.numeric(as.character(filter(UNOSpost_sort, quarter==i)$quarter)),
 as.numeric(as.character(filter(UNOSpost_sort, quarter==i)$pstatus2)),
 update_int_slope_predprob))
 names(StrategyD_1_predmat) <- c("quarter", "pstatus2", "predprob")

 # Store this data in the "master" list for future use
 StrategyD_1_predmat_master[[i]] <- StrategyD_1_predmat


 # Compute C-statistic in quarter i using Harrell's rms package. See:
 # https://stats.stackexchange.com/questions/145566/how-to-calculate-area-under-the-curve-auc-or-the-c-statistic-by-hand

 AUC_StrategyD_1[i] <- as.numeric(rcorr.cens(StrategyD_1_predmat$predprob, StrategyD_1_predmat$pstatus2))[1]


 # Compute the Mean Absolute Error (MAE) in quarter i
 MAE_StrategyD_1[i] <- mean(abs(StrategyD_1_predmat$predprob-StrategyD_1_predmat$pstatus2), na.rm=TRUE)


 # Compute Brier Score in quarter i
 Brier_StrategyD_1[i] <- mean((StrategyD_1_predmat$predprob-StrategyD_1_predmat$pstatus2)^2, na.rm=TRUE)


}


# For first 1 quarter, use same results as NEVER UPDATE (Strategy A)
for (i in 1){
 AUC_StrategyD_1[i] <- AUC_StrategyA[i]
 MAE_StrategyD_1[i] <- MAE_StrategyA[i]
 Brier_StrategyD_1[i] <- Brier_StrategyA[i]
}

# Convert the predmat list into a dataframe
StrategyD_1_predmat_allupdates <- do.call(rbind.data.frame, StrategyD_1_predmat_master)

# For first 1 quarter, use the StrategyA_predmat data
StrategyD_1_predmat_all <- rbind(StrategyA_predmat[which(StrategyA_predmat$quarter<2),],
 StrategyD_1_predmat_allupdates)

# For first 1 quarter, use the StrategyA_lm_master (i.e., baseline prediction model)
 StrategyD_1_lm_master[[1]] <- StrategyA_lm_master[[1]]

### Recalibrate the intercept & slope every quarter

Here, we recalibrate the intercept and slope of the model every quarter using the prior quarter of data. To do so, we follow the approach outlined by Vergouwe et al. 2017 – namely, we fit a logistic regression model in the update sample (in this case, the most recent quarter of data) with the linear predictor from the original (baseline) model as the only covariate. The slope of the updated model then serves as a “scaling factor” which rescales the original slope.

#############################################################################################
# Strategy E_1: Recalibrate intercept & slope every quarter using the prior quarter of data #
#############################################################################################

# Fit baseline prediction model using the UNOSpre dataframe
 baseline_int_slope_model <- glm(pstatus2 ~ agespline + #age
 as.numeric(as.character(ci_bin)) +
 as.numeric(as.character(calc_vent_use_bin)) +
 end_creat +
 as.numeric(as.character(creatinc2_flag)) +
 dx_group +
 as.numeric(as.character(func_stat_trr_bin)) +
 calc_o2 +
 calc_o2*dx_group + # o2 differs for groups B, C, D
 walkspline, #calc_six_min_walk,
 data=UNOSpre_sort,
 family="binomial")


# Get the linear predictor of baseline_int_slope_model in the full UNOSpost_sort data and save as new variable so that we can apply recalibrated model
UNOSpost_sort$lp_baseline <- predict(baseline_int_slope_model, UNOSpost_sort, type="link")


## Perform the remaining steps in a loop

for (i in 2:length(quarter_post)){ # Exclude baseline period

 # Recalibrate both the intercept and slope using the prior quarter of data by fitting a logistic
 # regression model with the linear predictor from the baseline model as the only covariate
 update_int_slope_model <- glm(pstatus2 ~ lp_baseline, # linear predictor from baseline model
 data=filter(UNOSpost_sort, quarter==(i-1)),
 family="binomial")

 # Store summary of recalibrated model at each quarter in master list for future reference
 StrategyE_1_lm_master[[i]] <- summary(update_int_slope_model)

 # Obtain predicted probabilities for the next quarter
 update_int_slope_predprob <- predict(update_int_slope_model,
 filter(UNOSpost_sort, quarter==i),
 type="response")

 # Create data frame with observed and predicted probabilities
 StrategyE_1_predmat <- as.data.frame(cbind(as.numeric(as.character(filter(UNOSpost_sort, quarter==i)$quarter)),
 as.numeric(as.character(filter(UNOSpost_sort, quarter==i)$pstatus2)),
 update_int_slope_predprob))
 names(StrategyE_1_predmat) <- c("quarter", "pstatus2", "predprob")

 # Store this data in the "master" list for future use
 StrategyE_1_predmat_master[[i]] <- StrategyE_1_predmat


 # Compute C-statistic in quarter i using Harrell's rms package. See:
 # https://stats.stackexchange.com/questions/145566/how-to-calculate-area-under-the-curve-auc-or-the-c-statistic-by-hand

 AUC_StrategyE_1[i] <- as.numeric(rcorr.cens(StrategyE_1_predmat$predprob, StrategyE_1_predmat$pstatus2))[1]


 # Compute the Mean Absolute Error (MAE) in quarter i
 MAE_StrategyE_1[i] <- mean(abs(StrategyE_1_predmat$predprob-StrategyE_1_predmat$pstatus2), na.rm=TRUE)


 # Compute Brier Score in quarter i
 Brier_StrategyE_1[i] <- mean((StrategyE_1_predmat$predprob-StrategyE_1_predmat$pstatus2)^2, na.rm=TRUE)


}


# For first 1 quarter, use same results as NEVER UPDATE (Strategy A)
for (i in 1){
 AUC_StrategyE_1[i] <- AUC_StrategyA[i]
 MAE_StrategyE_1[i] <- MAE_StrategyA[i]
 Brier_StrategyE_1[i] <- Brier_StrategyA[i]
}

# Convert the predmat list into a dataframe
StrategyE_1_predmat_allupdates <- do.call(rbind.data.frame, StrategyE_1_predmat_master)

# For first 1 quarter, use the StrategyA_predmat data
StrategyE_1_predmat_all <- rbind(StrategyA_predmat[which(StrategyA_predmat$quarter<2),],
 StrategyE_1_predmat_allupdates)

# For first 1 quarter, use the StrategyA_lm_master (i.e., baseline prediction model)
StrategyE_1_lm_master[[1]] <- StrategyA_lm_master[[1]]

### Recalibrate the intercept every quarter (“recalibration-in-the-large”)

Here, we recalibrate the intercept of the model every quarter using the prior quarter of data. To do so, we follow the approach outlined by Vergouwe et al. 2017 – namely, we fit a logistic regression model in the update sample (in this case, the most recent quarter of data) with the intercept as a free parameter and the linear predictor from the original (baseline) model as an offset term. This approach forces the slope of the updated model to equal 1.

#####################################################################################
# Strategy F_1: Recalibrate intercept every quarter using the prior quarter of data #
#####################################################################################

# Fit baseline prediction model using the UNOSpre dataframe
 baseline_int_slope_model <- glm(pstatus2 ~ agespline + #age
 as.numeric(as.character(ci_bin)) +
 as.numeric(as.character(calc_vent_use_bin)) +
 end_creat +
 as.numeric(as.character(creatinc2_flag)) +
 dx_group +
 as.numeric(as.character(func_stat_trr_bin)) +
 calc_o2 +
 calc_o2*dx_group + # o2 differs for groups B, C, D
 walkspline, #calc_six_min_walk,
 data=UNOSpre_sort,
 family="binomial")


# Get the linear predictor of baseline_int_slope_model in the full UNOSpost_sort data and save as new variable so that we can apply recalibrated model
UNOSpost_sort$lp_baseline <- predict(baseline_int_slope_model, UNOSpost_sort, type="link")


## Perform the remaining steps in a loop

for (i in 2:length(quarter_post)){ # Exclude baseline period

 # Recalibrate the intercept using the prior quarter of data by fitting a logistic regression model with the intercept as
 # a free parameter and the linear predictor from the baseline model as an offset term (to force the slope to equal 1)
 update_int_slope_model <- glm(pstatus2 ~ 1,
 data=filter(UNOSpost_sort, quarter==(i-1)),
 family="binomial",
 offset=lp_baseline)

 # Store summary of recalibrated model at each quarter in master list for future reference
 StrategyF_1_lm_master[[i]] <- summary(update_int_slope_model)

 # Obtain predicted probabilities for the next quarter
 update_int_slope_predprob <- predict(update_int_slope_model,
 filter(UNOSpost_sort, quarter==i),
 type="response")

 # Create data frame with observed and predicted probabilities
 StrategyF_1_predmat <- as.data.frame(cbind(as.numeric(as.character(filter(UNOSpost_sort, quarter==i)$quarter)),
 as.numeric(as.character(filter(UNOSpost_sort, quarter==i)$pstatus2)),
 update_int_slope_predprob))
 names(StrategyF_1_predmat) <- c("quarter", "pstatus2", "predprob")

 # Store this data in the "master" list for future use
 StrategyF_1_predmat_master[[i]] <- StrategyF_1_predmat


 # Compute C-statistic in quarter i using Harrell's rms package. See:
 # https://stats.stackexchange.com/questions/145566/how-to-calculate-area-under-the-curve-auc-or-the-c-statistic-by-hand

 AUC_StrategyF_1[i] <- as.numeric(rcorr.cens(StrategyF_1_predmat$predprob, StrategyF_1_predmat$pstatus2))[1]


 # Compute the Mean Absolute Error (MAE) in quarter i
 MAE_StrategyF_1[i] <- mean(abs(StrategyF_1_predmat$predprob-StrategyF_1_predmat$pstatus2), na.rm=TRUE)

 # Compute the Brier Score in quarter i
 Brier_StrategyF_1[i] <- mean((StrategyF_1_predmat$predprob-StrategyF_1_predmat$pstatus2)^2, na.rm=TRUE)


}


# For first 1 quarter, use same results as NEVER UPDATE (Strategy A)
for (i in 1){
 AUC_StrategyF_1[i] <- AUC_StrategyA[i]
 MAE_StrategyF_1[i] <- MAE_StrategyA[i]
 Brier_StrategyF_1[i] <- Brier_StrategyA[i]
}

# Convert the predmat list into a dataframe
StrategyF_1_predmat_allupdates <- do.call(rbind.data.frame, StrategyF_1_predmat_master)

# For first 1 quarter, use the StrategyA_predmat data
StrategyF_1_predmat_all <- rbind(StrategyA_predmat[which(StrategyA_predmat$quarter<2),],
 StrategyF_1_predmat_allupdates)

# For first 1 quarter, use the StrategyA_lm_master (i.e., baseline prediction model)
StrategyF_1_lm_master[[1]] <- StrategyA_lm_master[[1]]

### Update model every quarter via closed testing procedure (Vergouwe et al. 2017)

Here, we apply the closed testing procedure by Vergouwe et al. 2017 to each quarter of data to select which of the previous four models (never updated [A], revised/completely refit model [D_1], recalibrated intercept and slope [E_1], and recalibrated-in-the-large [F_1]) to use during the next quarter. This procedure involves sequential likelihood ratio tests:

A. Conduct an LRT of the revised model [D_1] vs. the baseline model [A] using a significance level of alpha and p+1 degrees of freedom
 - If not significant, keep baseline model [A]; STOP.
 - If significant, CONTINUE.

B. Conduct an LRT of the revised model [D_1] vs. the model whose intercept was recalibrated [F_1] using a significance level of alpha and p degrees of freedom
 - If not significant, keep [F_1]; STOP.
 - If significant, CONTINUE.

C. Conduct an LRT of the revised model [D_1] vs. the model whose intercept and slope were recalibrated [E_1] using a significance level of alpha and df = p-1
 - If not significant, keep [E_1]; STOP.
 - If significant, keep [D_1]; END OF PROCEDURE.

The code below has been adapted from the appendix of Vergouwe et al. 2017

##############################################################################################
# Strategy V_1: Apply Closed Testing Procedure every quarter using the prior quarter of data #
##############################################################################################

# Fit baseline prediction model using the UNOSpre dataframe
 baseline_int_slope_model <- glm(pstatus2 ~ agespline + #age
 as.numeric(as.character(ci_bin)) +
 as.numeric(as.character(calc_vent_use_bin)) +
 end_creat +
 as.numeric(as.character(creatinc2_flag)) +
 dx_group +
 as.numeric(as.character(func_stat_trr_bin)) +
 calc_o2 +
 calc_o2*dx_group + # o2 differs for groups B, C, D
 walkspline, #calc_six_min_walk,
 data=UNOSpre_sort,
 family="binomial")


# Get the linear predictor of baseline_int_slope_model in the full UNOSpost_sort data and save as new variable so that we can apply recalibrated model
UNOSpost_sort$lp_baseline <- predict(baseline_int_slope_model, UNOSpost_sort, type="link")

# Initialize index_test vector to store which model the closed testing procedure selected at each quarter
index_test_vec <- rep(NA, max(quarter_post))

## Perform the remaining steps in a loop

for (i in 2:length(quarter_post)){ # Exclude baseline period

 #---------------------------------#
 # Fit the candidate update models #
 #---------------------------------#

 # Refit the model (model revision)
 revised_int_slope_model <- glm(pstatus2 ~ agespline + #age
 as.numeric(as.character(ci_bin)) +
 as.numeric(as.character(calc_vent_use_bin)) +
 end_creat +
 as.numeric(as.character(creatinc2_flag)) +
 dx_group +
 as.numeric(as.character(func_stat_trr_bin)) +
 calc_o2 +
 calc_o2*dx_group + # o2 differs for groups B, C, D
 walkspline, #calc_six_min_walk,
 data=filter(UNOSpost_sort, quarter==(i-1)),
 family="binomial")

 # Recalibrate intercept and slope (recalibration)
 recalibrated_int_slope_model <- glm(pstatus2 ~ lp_baseline,
 data=filter(UNOSpost_sort, quarter==(i-1)),
 family="binomial")

 # Recalibrate the intercept (recalibration-in-the-large)
 recalibrated_int_model <- glm(pstatus2 ~ 1,
 data=filter(UNOSpost_sort, quarter==(i-1)),
 family="binomial",
 offset=lp_baseline)


 #---------------------------------------------------------------------#
 # Compute the linear predictors & log-likelihoods of candidate models #
 # using the same data in which they were developed (here, i-1) #
 #---------------------------------------------------------------------#

 update_samp <- filter(UNOSpost_sort, quarter==(i-1))

 # Compute linear predictor and log-likelihood of baseline model
 lp_original <- predict(baseline_int_slope_model, update_samp, type="link")
 ll_original <- sum(update_samp$pstatus2 * lp_original - log(1 + exp(lp_original)))

 # Compute linear predictor and log-likelihood of revised model
 lp_revised <- predict(revised_int_slope_model, update_samp, type="link")
 ll_revised <- sum(update_samp$pstatus2 * lp_revised - log(1 + exp(lp_revised)))

 # Compute linear predictor and log-likelihood of recalibrated intercept & slope model
 lp_recalibrated_int_slope <- predict(recalibrated_int_slope_model, update_samp, type="link")
 ll_recalibrated_int_slope <- sum(update_samp$pstatus2 * lp_recalibrated_int_slope - log(1 + exp(lp_recalibrated_int_slope)))

 # Compute linear predictor and log-likelihood of recalibrated intercept model
 lp_recalibrated_int <- predict(recalibrated_int_model, update_samp, type="link")
 ll_recalibrated_int <- sum(update_samp$pstatus2 * lp_recalibrated_int - log(1 + exp(lp_recalibrated_int)))


 #---------------------------------------------------------------#
 # Compute the difference in log-likelihoods of candidate models #
 #---------------------------------------------------------------#

 dev_original <- -2*(ll_original - ll_revised) # Test 1: Revised model vs. baseline model
 dev_recalibrated_int <- -2*(ll_recalibrated_int - ll_revised) # Test 2: Revised model vs. recalibrated intercept model
 dev_recalibrated_int_slope <- -2*(ll_recalibrated_int_slope - ll_revised) # Test 3: Revised model vs. recalibrated intercept & slope model


 #-------------------------------------------------------------------#
 # Conduct the LRT to see if difference in model fit was significant #
 #-------------------------------------------------------------------#

 # When calculating degrees of freedom, need to subtract 1 from the length command to ensure that intercept is not included
 test1 <- (1 - pchisq(dev_original, df=(length(revised_int_slope_model$coefficients)-1)+1)) < 0.05
 test2 <- (1 - pchisq(dev_recalibrated_int, df=(length(revised_int_slope_model$coefficients)-1))) < 0.05
 test3 <- (1 - pchisq(dev_recalibrated_int_slope, df=(length(revised_int_slope_model$coefficients)-1)-1)) < 0.05


 #-----------------------------------#
 # Determine which model is selected #
 #-----------------------------------#

 # 1 = Original model, 2 = Model with updated intercept, 3 = Recalibrated model, 4 = Revised model

 test_original <- 1 * (!test1)
 test_recalibrated_int <- 2 * ((test1) & (!test2))
 test_recalibrated_int_slope <- 3 * ((test1) & (test2) & (!test3))
 test_revised <- 4 * ((test1) & (test2) & (test3))

 index_test <- (test_original + test_recalibrated_int + test_recalibrated_int_slope + test_revised)
 index_test_vec[i] <- index_test

 #------------------------------------#
 # Apply chosen model to next quarter #
 #------------------------------------#

 if (index_test_vec[i]==1){ # Keep baseline model

 # Store summary of chosen model at each quarter in master list for future reference
 StrategyV_1_lm_master[[i]] <- summary(baseline_int_slope_model)

 # Obtain predicted probabilities for the next quarter
 update_int_slope_predprob <- predict(baseline_int_slope_model,
 filter(UNOSpost_sort, quarter==i),
 type="response")

 }


 else if (index_test_vec[i]==2){ # Use recalibrated_int model (recalibrated-in-the-large)

 # Store summary of chosen model at each quarter in master list for future reference
 StrategyV_1_lm_master[[i]] <- summary(recalibrated_int_model)

 # Obtain predicted probabilities for the next quarter
 update_int_slope_predprob <- predict(recalibrated_int_model,
 filter(UNOSpost_sort, quarter==i),
 type="response")

 # Use the recalibrated_int model as the new baseline model
 baseline_int_slope_model <- recalibrated_int_model
 UNOSpost_sort$lp_baseline <- predict(baseline_int_slope_model, UNOSpost_sort, type="link")
 }

 else if (index_test_vec[i]==3){ # Use recalibrated_int_slope model (recalibrated intercept & slope)

 # Store summary of chosen model at each quarter in master list for future reference
 StrategyV_1_lm_master[[i]] <- summary(recalibrated_int_slope_model)

 # Obtain predicted probabilities for the next quarter
 update_int_slope_predprob <- predict(recalibrated_int_slope_model,
 filter(UNOSpost_sort, quarter==i),
 type="response")

 # Use the recalibrated_int_slope model as the new baseline model
 baseline_int_slope_model <- recalibrated_int_slope_model
 UNOSpost_sort$lp_baseline <- predict(baseline_int_slope_model, UNOSpost_sort, type="link")
 }

 else if (index_test_vec[i]==4){ # Use revised (completely refitted) model

 # Store summary of chosen model at each quarter in master list for future reference
 StrategyV_1_lm_master[[i]] <- summary(revised_int_slope_model)

 # Obtain predicted probabilities for the next quarter
 update_int_slope_predprob <- predict(revised_int_slope_model,
 filter(UNOSpost_sort, quarter==i),
 type="response")

 # Use the revised model as the new baseline model
 baseline_int_slope_model <- revised_int_slope_model
 UNOSpost_sort$lp_baseline <- predict(baseline_int_slope_model, UNOSpost_sort, type="link")
 }


 # Create data frame with observed and predicted probabilities from chosen model
 StrategyV_1_predmat <- as.data.frame(cbind(as.numeric(as.character(filter(UNOSpost_sort, quarter==i)$quarter)),
 as.numeric(as.character(filter(UNOSpost_sort, quarter==i)$pstatus2)),
 update_int_slope_predprob))
 names(StrategyV_1_predmat) <- c("quarter", "pstatus2", "predprob")

 # Store this data in the "master" list for future use
 StrategyV_1_predmat_master[[i]] <- StrategyV_1_predmat


 # Compute C-statistic in quarter i using Harrell's rms package. See:
 # https://stats.stackexchange.com/questions/145566/how-to-calculate-area-under-the-curve-auc-or-the-c-statistic-by-hand

 AUC_StrategyV_1[i] <- as.numeric(rcorr.cens(StrategyV_1_predmat$predprob, StrategyV_1_predmat$pstatus2))[1]


 # Compute the Mean Absolute Error (MAE) in quarter i
 MAE_StrategyV_1[i] <- mean(abs(StrategyV_1_predmat$predprob-StrategyV_1_predmat$pstatus2), na.rm=TRUE)


 # Compute the Brier Score in quarter i
 Brier_StrategyV_1[i] <- mean((StrategyV_1_predmat$predprob-StrategyV_1_predmat$pstatus2)^2, na.rm=TRUE)


}


# For first 1 quarter, use same results as NEVER UPDATE (Strategy A)
for (i in 1){
 AUC_StrategyV_1[i] <- AUC_StrategyA[i]
 MAE_StrategyV_1[i] <- MAE_StrategyA[i]
 Brier_StrategyV_1[i] <- Brier_StrategyA[i]
}

# Convert the predmat list into a dataframe
StrategyV_1_predmat_allupdates <- do.call(rbind.data.frame, StrategyV_1_predmat_master)

# For first 1 quarter, use the StrategyA_predmat data
StrategyV_1_predmat_all <- rbind(StrategyA_predmat[which(StrategyA_predmat$quarter<2),],
 StrategyV_1_predmat_allupdates)

# For first 1 quarter, use the StrategyA_lm_master (i.e., baseline prediction model)
StrategyV_1_lm_master[[1]] <- StrategyA_lm_master[[1]]

# Rename index_text_vec so that we can use it later
index_test_vec_1q <- index_test_vec

#

# **References**

1. Vergouwe Y, Nieboer D, Oostenbrink R, Debray TPA, Murray GD, Kattan MW, Koffijberg H, Moons KGM, Steyerberg EW. A closed testing procedure to select an appropriate method for updating prediction models. *Statistics in Medicine* 2017; 36: 4529-4539.

2. Steyerberg EW. Updating for a New Setting. In: Clinical Prediction Models. A Practical Approach to Development, Validation, and Updating. New York: Springer Science+Business Media, LLC. 2010.

3. Jenkins DA, Sperrin M, Margin GP, Peek N. Dynamic models to predict health outcomes: current status and methodological challenges. *Diagnostic and Prognostic Research* 2018; 2:23.

4. Steyerberg EW, Vergouwe Y. Towards better clinical prediction models: seven steps for development and an ABCD for validation. *European Heart Journal* 2014; 35: 1925-1931.

5. Van Calster B, Nieboer D, Vergouwe Y, De Cock B, Pencina MJ, Steyerberg EW. A calibration hierarchy for risk models was defined: from utopia to empirical data. *Journal of Clinical Epidemiology* 2016; 74: 167-176.

6. Su T, Jaki T, Hickey GL, Buchan I, Sperrin M. A review of statistical updating methods for clinical prediction models. *Statistical Methods in Medical Research* 2018; 27(1): 185-197.

7. Cox DR. Two further applications of a model for binary regression. *Miscellanea*. 1958.

8. Miller ME, Langefeld CD, Tierney WM, Hui SL, McDonald CJ. Validation of Probabilistic Predictions. *Medical Decision Making* 1993; 13: 49-58.
